# Genomic Relationships and Speciation Times of Human, Chimpanzee, and Gorilla Inferred from a Coalescent Hidden Markov Model

**DOI:** 10.1371/journal.pgen.0030007

**Published:** 2007-02-23

**Authors:** Asger Hobolth, Ole F Christensen, Thomas Mailund, Mikkel H Schierup

**Affiliations:** 1 Bioinformatics Research Center, North Carolina State University, Raleigh, North Carolina, United States of America; 2 Bioinformatics Research Center, University of Aarhus, Aarhus, Denmark; 3 Department of Statistics, University of Oxford, Oxford, United Kingdom; Broad Institute of MIT and Harvard, United States of America

## Abstract

The genealogical relationship of human, chimpanzee, and gorilla varies along the genome. We develop a hidden Markov model (HMM) that incorporates this variation and relate the model parameters to population genetics quantities such as speciation times and ancestral population sizes. Our HMM is an analytically tractable approximation to the coalescent process with recombination, and in simulations we see no apparent bias in the HMM estimates. We apply the HMM to four autosomal contiguous human–chimp–gorilla–orangutan alignments comprising a total of 1.9 million base pairs. We find a very recent speciation time of human–chimp (4.1 ± 0.4 million years), and fairly large ancestral effective population sizes (65,000 ± 30,000 for the human–chimp ancestor and 45,000 ± 10,000 for the human–chimp–gorilla ancestor). Furthermore, around 50% of the human genome coalesces with chimpanzee after speciation with gorilla. We also consider 250,000 base pairs of X-chromosome alignments and find an effective population size much smaller than 75% of the autosomal effective population sizes. Finally, we find that the rate of transitions between different genealogies correlates well with the region-wide present-day human recombination rate, but does not correlate with the fine-scale recombination rates and recombination hot spots, suggesting that the latter are evolutionarily transient.

## Author Summary

Primate evolution is a central topic in biology and much information can be obtained from DNA sequence data. A key parameter is the time “when we became human,” i.e., the time in the past when descendents of the human–chimp ancestor split into human and chimpanzee. Other important parameters are the time in the past when descendents of the human–chimp–gorilla ancestor split into descendents of the human–chimp ancestor and the gorilla ancestor, and population sizes of the human–chimp and human–chimp–gorilla ancestors. To estimate speciation times and ancestral population sizes we have developed a new methodology that explicitly utilizes the spatial information in contiguous genome alignments. Furthermore, we have applied this methodology to four long autosomal human–chimp–gorilla–orangutan alignments and estimated a very recent speciation time of human and chimp (around 4 million years) and ancestral population sizes much larger than the present-day human effective population size. We also analyzed X-chromosome sequence data and found that the X chromosome has experienced a different history from that of autosomes, possibly because of selection.

## Introduction

The recent evolutionary history of the human species can be investigated by comparative approaches using the genomes of the great apes: chimpanzee, gorilla, and orangutan [1]. Nucleotide differences, accumulated by fixation of mutations, carry a wealth of information on important issues such as speciation times, properties of ancestral species (e.g., population sizes), and how speciation occurred [2–5]. Genes or genomic fragments with unusual patterns of nucleotide differences and divergence may have been under strong natural selection during recent evolution of the human species [6]. Sequence analyses can also aid interpretations of the incomplete primate fossil records [7] and aid assignment of dated fossils to evolutionary lineages. For instance, it is still debated whether the Millennium man, Orrorin tugenensis [8,9], which has been dated to 6 million years (Myr) ago, and Sahelanthropus tchadensis [10–12], which has been dated to 6–7 Myr ago, belong to the human lineage or the human–chimp (HC) lineage.

**Figure 1:**
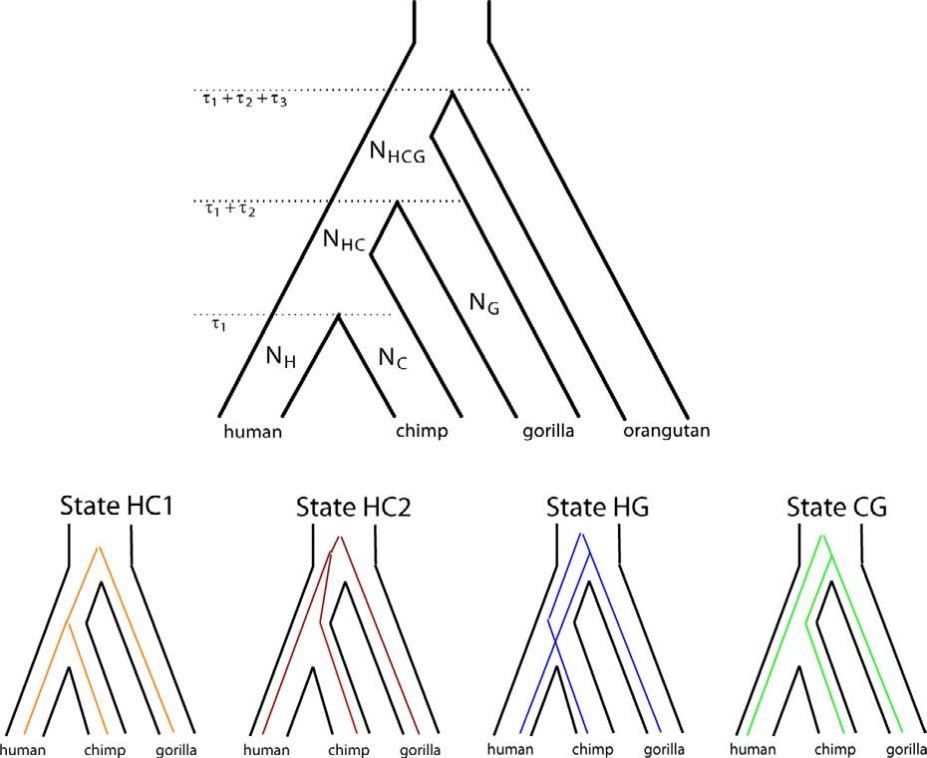
Genetic and Species Relationships May Differ Top: Genealogical relationship of human, chimpanzee, gorilla, and orangutan. Speciation times are denoted *τ*_1,_ *τ*_1_ + *τ*_2_, and *τ*_1_ + *τ*_2_ + *τ*_3_. Population sizes of human, chimpanzee, and gorilla are denoted *N*_H_, *N*_C_, and *N*_G_, while the HC and HCG ancestral population sizes are denoted *N*_HC_ and *N*_HCG_. Bottom: Each of the four hidden states in the coal-HMM corresponds to a particular phylogenetic tree. In state HC1, human and chimpanzee coalesce before speciation of human, chimpanzee, and gorilla, i.e., before *τ*_1_ + *τ*_2_. In states HC2, HG, and CG, human, chimpanzee, and gorilla coalesce after speciation of the three species, i.e., after *τ*_1_ + *τ*_2_. In HC2, the human and chimpanzee lineages coalesce first, and then the HC lineage coalesces with gorilla. In state HG, human and gorilla coalesce first, and in state CG, chimpanzee and gorilla coalesce first. The hidden phylogenetic states cannot be observed from present-day sequence data, but they can be decoded using the coal-HMM methodology.

Comparative analyses of multiple alignments of small fragments of human, chimpanzee, gorilla, and orangutan sequence have revealed that the human genome is more similar to the gorilla genome than to the chimpanzee genome for a considerable fraction of single genes [2,13–15]. Such a conflict between species and gene genealogy is expected if the time span between speciation events is small measured in the number of 2*N* generations, where *N* is the effective population of the ancestral species (see Figure 1). In that case, *N* can be estimated from the proportion of divergent genealogies if one assumes that speciation is an instantaneous event. Indeed, this has been done in several studies that find a HC ancestral effective population size *N*_HC_ of 2–10 times the human present-day effective population size *N*_H_ = 10,000 [13,14,16–18]. Recently, Patterson et al. [2] studied a very large number of small human–chimp–gorilla–orangutan–macaque alignments. They found, in agreement with O'hUigin et al. [15], that a large proportion of sites supporting alternative genealogies are caused by hypermutability and that the fraction of the genome with alternative genealogies therefore has been overestimated in previous studies. After using a statistical correction for substitution rate heterogeneity, Patterson et al. [2] found that the variance in coalescence times is too large to be accounted for by instant speciation and a large ancestral effective population size, and that the speciation process therefore must have been complex. Particularly, the X chromosome shows a deviant pattern, which also led them to conclude that HC gene flow ceased and final speciation occurred as recently as 4 Myr ago. This date is generally believed to be the most recent time compatible with the fossil record, if the Millennium man and *Sahelanthropus* are not on the human lineage.

Whole genome sequences of gorilla and orangutan will soon supplement the already available whole genome sequences of human and chimpanzee [19]. These four genomes are so closely related that alignments of large contiguous parts of the genomes can be constructed. Analysis of such large fragments is challenging because different parts of the alignment will have different evolutionary histories (and thus different genealogies, see Figure 1) because of recombination [14,20]. Ideally, one would like to infer the genealogical changes directly from the data and then analyze each type of genealogy separately. A natural approach to this challenge is to move along the alignment, and simultaneously compute the probabilities of different relationships and speciation times. While recombination has been considered in previous likelihood models [14], the spatial information along the alignment has largely been ignored.

**Figure 2:**
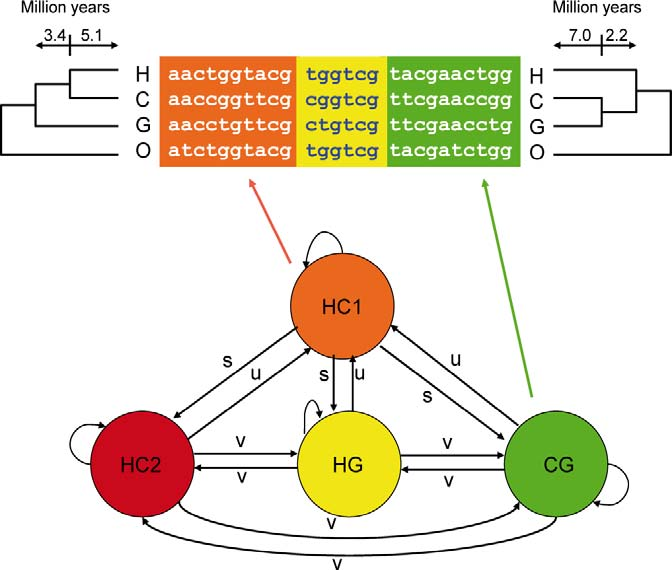
Graphical Representation of Coal-HMM The coal-HMM divides a multiple alignment into four types of segments corresponding to the four phylogenetic states HC1, HC2, HG, and CG. The probability of making a transition from state HC1 to any of the other states is *s,* and the probability of a transition from any of the HC2, HG, or CG states to state HC1 is *u*. Transitions between the HC2, HG, and CG states have probability *v*. The indicated branch lengths of the phylogenetic trees from state HC1 and state CG are divergence times estimated from Target 1. The branch lengths of the phylogenetic trees corresponding to state HC2 and HG are the same as the branch lengths of state CG.

In this paper we describe a hidden Markov model (HMM) that allows the presence of different genealogies along large multiple alignments. The hidden states are different possible genealogies (labeled HC1, HC2, HG, and CG in Figures 1 and 2). Parameters of the HMM include population genetics parameters such as the HC and human–chimp–gorilla (HCG) ancestral effective population sizes, *N*_HC_ and *N*_HCG_, and speciation times *τ*_1_ and *τ*_2_ (see Figure 1). We therefore name our approach a coalescent HMM (coal-HMM). The statistical framework of HMMs yields parameter estimates with associated standard errors, and posterior probabilities of hidden states [21–23]. We show by simulation studies that the coal-HMM recovers parameters from the coalescence with recombination process, and we apply the coal-HMM to five long contiguous human–chimp–gorilla–orangutan (HCGO) alignments obtained from the NIH Intramural Sequencing Center comparative sequencing program (Targets 1, 106, 121, and 122 on four different autosomes and Target 46 on the X chromosome). We consistently find very recent estimates of HC speciation times and a large variance in the time to common ancestry along the genome. Similar to Patterson et al. [2], we find that the X chromosome has a smaller effective population size than expected. The mapping of genealogical states further allows us to correlate transitions in genealogies with properties of the genome, and here we focus on fine-scale [24] and region-wide [25] recombination rate estimates.

## Results

**Figure 3:**
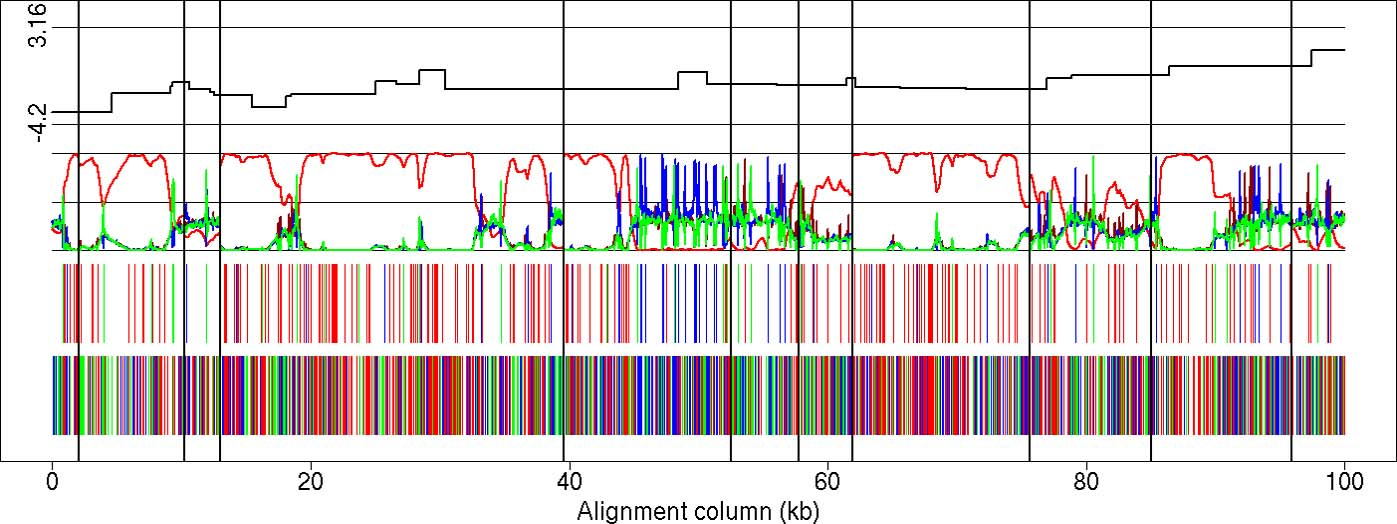
Inferred Genealogies from Real Data (From bottom to top) Analysis of the first 100 kb from Target 1. (Bottom) Site information without outgroup: sites shared by HC in red, by HG in blue, and by CG in green (compare first, third, and last columns in Table 1). (Second from bottom) Site information with outgroup: sites strongly supporting states HC1 or HC2 in red, HG in blue, and CG in green (compare second, third, and last columns in Table 1). (Third from bottom) Posterior probabilities: Coloring as in second from bottom, except that state HC2 is dark red. (Top) Fine-scale recombination rate estimates (log scale). The vertical lines mark subdivisions of the multiple alignment due to more than 50-base-pair deletions in one species (see “Data” in Materials and Methods).

A visual impression of the preferred phylogenetic state along the alignment is obtained by dividing the alignment sites according to how they partition the species (Table 1). For example, sites where human and gorilla have the same base pair, which is different from chimpanzee, suggest a human–gorilla (HG) grouping. The HG grouping is further supported if the outgroup (orangutan) has the same base pair as the chimpanzee. In Figure 3, we show the first 100 kb from Target 1. The preferred topologies along the alignment are shown in the third (support with outgroup) and fourth (support without outgroup) panels of the figure, from top to bottom.

**Table 1:**
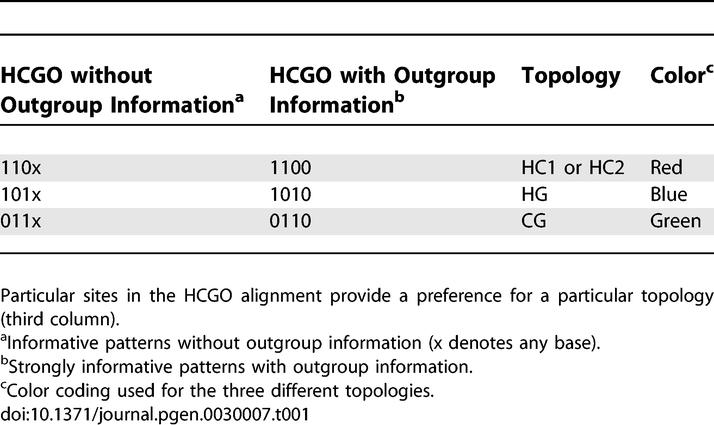
Divergent Sites Provide Information about Genealogy

The posterior probabilities of the phylogenetic states are shown in the panel second from the top in Figure 3. Similar figures for all targets in 100-kb blocks can be found in Figures S1–S5. The density and character of the strongly informative sites in the third panel and singletons in the fourth panel largely determine the inferred states along the alignment. State HC1 is generally the preferred state; this state is often strongly supported over contiguous portions of the alignment and typically spans several kilobases. The alternative states HC2, HG, and CG (chimp–gorilla) are less strongly supported and typically cover very short segments. The upper panel shows the fine-scale recombination rates determined from human polymorphism data [24]. No clear association is observed between transitions in the HMM and these rates, e.g., recombination hotspots are not concentrated in regions of rapidly changing genealogies (for a formal statistical test, see Text S1).

**Figure 4:**
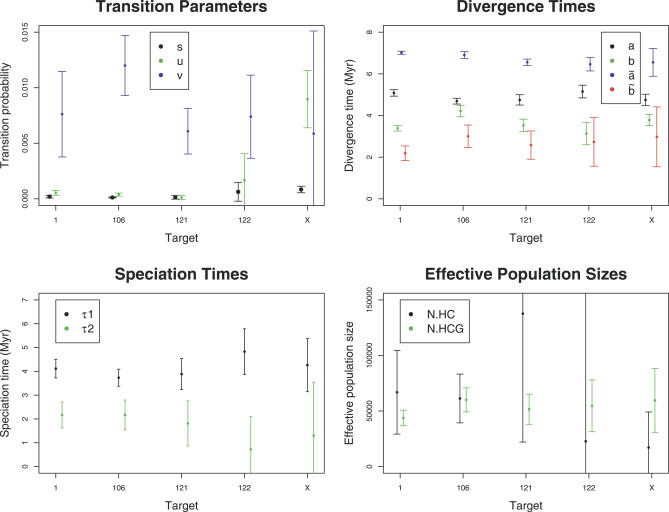
Parameter Estimates from Coal-HMM Analysis of Five Targets Estimates with associated standard errors of the HMM and population genetics parameters for the five targets. Top left plot shows the HMM transition rates, top right plot the genetic divergence times in million years (assuming orangutan divergence 18 Myr ago), bottom left plot the speciation times in million years, and bottom right plot the ancestral effective population sizes, again assuming orangutan divergence of 18 Myr and a generation time of 25 y for all species throughout the HCGO divergence.

Parameter estimates with standard errors for each target are shown in Figure 4. Assuming orangutan divergence 18 Myr ago [18], speciation time of human and chimpanzee is consistently around 4 Myr with small standard errors except for the very short Target 122. The speciation times for the X chromosome are also in close agreement with the speciation times of the autosomes. The orangutan divergence assumed is the molecular divergence time, which may be much larger than the orangutan speciation time if the *N_HCGO_* effective population size was large. If another orangutan divergence time *Z* is preferred, then all our time estimates should be multiplied by *Z*/18.

The divergence times in Figure 4 are much higher than speciation times because of large effective population sizes in the ancestral species. We note that the effective population size of the HCG ancestor is more accurately determined than that of the HC ancestor, which may seem counterintuitive, suggesting that ancestral inference of certain quantities does not necessarily become increasingly difficult further back in time. Exactly the same pattern was found in simulations, suggesting that this is a true property of the process.

Target 1 was also analyzed after filtering out all putative CpG mutations. This reduces the number of polymorphic sites by 17% and removes relatively more of the sites supporting HG and CG groupings than sites supporting HC grouping (states HC1 and HC2), as expected if some of these sites are hypermutable. However, the removal of putative CpG sites does not change the estimated time in alternative states or effective population sizes and only slightly decreases the estimated HC speciation time. The time spent in the alternative states HC2, HG, and CG is also only slightly affected (Text S2). The visual impression of figures such as Figure 3 also remains the same after filtering out the hypermutable CpG sites. We also aligned Target 1 to a further outgroup, gibbon, in order to identify sites showing evidence for recurrent mutations resulting in implausible five-species site patterns (see also [2]). Removing these sites further reduces the proportion of strongly informative sites supporting the HG and CG states, but the proportion of time spent in each state, speciation times, and divergence time estimates are again only slightly affected after removal of these sites (see Text S2).

While estimates of the effective population sizes and speciation times do not differ significantly between the four targets, there are large differences in the average number of base pairs and the percentage of the alignment in state HC1 (Table 2). The average number of base pairs in state HC1 correlates well with the average recombination rate of the target estimated from pedigree data [25]. Thus, this broader scale recombination rate appears to be conserved over a longer time scale than the fine-scale recombination rate estimated from human diversity data, and it appears to be shared with chimpanzee and gorilla.

**Table 2:**
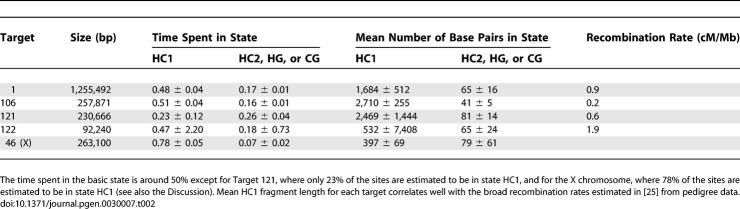
Mean Fragment Lengths for Basic State HC1 Correlate with Pedigree Recombination Rate

**Figure 5:**
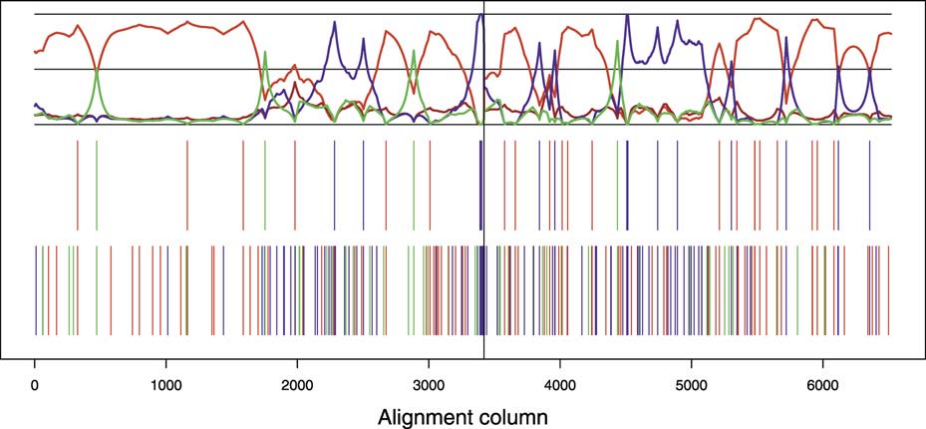
Inferred Genealogies along a 6-kb Region of the X Chromosome We observe several adjacent sites that support alternative state HG (blue lines), corresponding to coalescence of human and gorilla before coalescence with chimpanzee.

A coal-HMM analysis of more than 250 kb of X-chromosome sequence data used by [2] shows that 78% ± 5% of the alignment supports state HC1 (Figure 2). One of the deviant regions is shown in Figure 5, where a cluster of sites supporting a HG relationship is observed (figures for the whole target are available in Figure S5). Thus we find that not all of the X-chromosome data are consistent with state HC1 as previously suggested [2]. However, only a small fraction of the X-chromosome data support alternative states, and this is consistent with an effective population size of the X chromosome in the HC ancestor of approximately 17,000 (see Figure 4), which is much lower than expected assuming an effective population size of 75% that of the autosomes. Curiously, the HCG ancestor *N*_HCG_ for the X chromosome data is close to the expected (see Figure 4).

## Discussion

Studying the genealogical relationship of human, chimpanzee, and gorilla along their genomes makes it possible to assign genealogical relationships to segments of the genome with high posterior probabilities (Figures 3 and S1–S5). Long fragments of several kilobases supporting the basic state HC1 alternate with kilobase-long fragments that support the alternative states HC2, HG, and CG. Since alternative states imply coalescence further back in time, the ancestral material is expected to be broken up more by recombination in regions supporting these states. Thus, frequent changes among alternative states are predicted by coalescent theory, but this has usually not been explicitly considered in previous analyses. The picture of alternating genealogies can subsequently be correlated with genomic features such as specific genes suspected to be important in human evolution, and can be used to survey whole genomes for extraordinarily long segments indicative of selection and/or recent introgression. The complex speciation model suggested in Patterson et al. [2] can also be more closely investigated using extensions of our coal-HMM framework when longer contiguous alignments become available.

Important insights can already be gained from analyzing 1.9 million base pairs from four autosomal segments of the genome, and 0.25 million base pairs from the X chromosome. We consistently find that the speciation time of human and chimpanzee is close to the minimum of the range previously predicted (4 Myr) if we assume a human–orangutan divergence of 18 Myr. If the effective population size *N*_HCGO_ was large, then there is also a large variation in orangutan divergence. However, 18 Myr ago, the size of ancestral segments was very short (a few base pairs), so the variation in divergence times over kilobase-long fragments as studied here is expected to be small. The HCG speciation time is estimated to have occurred approximately 2 Myr earlier than the HC speciation time (i.e., 6 Myr ago). However, the divergences along the genome of human and chimpanzees are generally much older than 4 Myr, varying between 4 and 9 Myr. This can be explained solely by ancestral population sizes on the order of 50,000 in the HC and HCG ancestors, and one does not need to invoke a gradual speciation process with continued gene flow (introgression) to explain the autosomal data, as also noted by Innan and Watanabe [4] and Barton [5].

Our molecular dating estimates are generally in agreement with a large number of studies using different calibration points; Kumar et al. [26], Glazko and Nei [27], and even the classical study of Sarich and Wilson [28] found a molecular divergence of HC at 5–7 Myr, 6 Myr, and 5 Myr, respectively. Speciation, defined as the total cessation of gene flow, is necessarily more recent than these molecular dates, and our value of approximately 4 Myr agrees very well with the time suggested by Patterson et al. [2] for complete cessation of gene flow. It is also in agreement with the oldest fossils generally accepted to belong to the human lineage after the HC split. The autosomal analysis alone cannot be used to determine if the large variance in coalescence times of human and chimp along the genome is due to a large ancestral effective population size or due to prolonged speciation [5].

The present implementation of the coal-HMM assumes that, conditional on the genealogy, sites are independent and mutations can be described by a continuous time Markov chain. This assumption is violated for CpG dinucleotides, which are more prone to mutation due to methylation. The assumption is also violated if the mutation rate varies along the alignment, resulting in regions with highly variable sites that are subject to recurrent mutations. Recurrent mutations can be detected by adding a further outgroup [2,15]. We used a similar strategy as Patterson et al. [2] to mask CpG-induced variable sites and sites with clear evidence of recurrent mutation on the human–chimp–gorilla–orangutan–gibbon phylogeny. Our analyses show that removal of these sites does not affect our estimates of genealogies along the alignment, of the divergence times, or of the speciation times very much. We ascribe this robustness of the coal-HMM to the fact that the model uses information from the singleton sites, which are much more common than the strongly informative sites (recall Table 1). Indeed, we have calculated that the evidence for distinguishing between genealogical states of a strongly informative site corresponds to 5–7 singletons supporting the same state.

The 250,000 base pairs aligned on the X chromosome have a larger than expected fraction of base pairs in state HC1 (around 80%) if the long-term effective population size of the X chromosome is three-quarters that of the autosomes. There is strong evidence that a small segment on the X chromosome supports an alternative genealogy (state HG). However, the effective population size is reduced by much more than the expected 25% assuming instant speciation, equal contribution of sexes, and no selection. Non-equal contribution of sexes can at most reduce the effective population size to 50% that of autosomes (if females have much larger variance in reproductive success than males). Prolonged speciation and selection may both explain the discrepancy. The X chromosome, with its hemizygosity in males, is more exposed to selection, and this can be an argument for a different introgression history (in a prolonged speciation process) or for selection generally affecting the X chromosome more in the HC ancestor, thus reducing the effective population size more than on the autosomes. The observed fraction of 80% in state HC1 is expected when the effective population size of the X chromosome is approximately 35% that of the autosomes. Explicit modeling of introgression processes and the natural selection on the X chromosome, together with an extended coal-HMM (that explicitly models the length distribution of segments supporting different genealogies), may provide the means to test among these alternative explanations when more data become available.

Another feature of the human genome that can be explored using the coal-HMM is the evolutionary scale of variation in recombination rate. We observe a correlation between the region-wide recombination rate estimated from pedigrees and the average length of segments in state HC1, consistent with evolutionary conservation of these region-wide recombination rates. However, we see no clear correlation between the fine-scale recombination rate estimated from human polymorphism data and transitions to and from state HC1. We interpret this as additional evidence that recombination hot spots are very transient, since we are analyzing an even shorter time scale (at least half) than that used when comparing human and chimpanzee, where recombination hot spots do not appear to be shared [24].

When more data become available, the coal-HMM can be extended to investigate more specific hypotheses. Our simulations show that the coal-HMM provides a reasonable approximation to the much more complex coalescent-with-recombination process. Furthermore, we found that adding more parameters in the transition probability matrix did not improve the fit significantly with 1.9 million base pairs. With a 1,000-fold increase in data soon to arrive, a natural extension is to introduce more hidden states in the HMM to provide a more detailed approximation of the different coalescent times. When this is done we expect a better fit of segment sizes to the geometric distribution assumed by the coal-HMM. Importantly, having more data will make it possible to investigate changes in ancestral effective population sizes along the genome, thus making it possible to infer cases of ancestral selection in the HC ancestor and in the HCG ancestor, and opening up a promising field of ancestral species population genetics that can complement analyses based on *dn/ds* ratios. It will also be possible to explicitly test whether the effective population sizes of ancestral species *N*_HC_ and *N*_HCG_ have changed through time.

## Materials and Methods

### Coal-HMM.

Coal-HMMs provide the framework for analyses of genome alignments of human, gorilla, chimpanzee, and orangutan sequences. Coal-HMMs are similar to phylogenetic HMMs [29], but instead of partitioning the alignment into fragments undergoing different evolutionary processes because of functional properties (e.g., noncoding, exonic, and intronic regions), the alignment is partitioned into fragments of different evolutionary histories separated by recombination events. In our coal-HMM we consider recombination events that separate four different genealogies. The four genealogies are shown in Figure 1 and correspond to the hidden states of the model.

The transitions between the hidden states are modeled using a Markov chain with transition probability matrix *P*(·,·). We have primarily studied a transition matrix given by

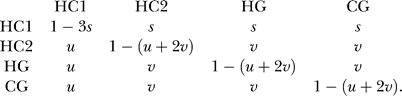



The stationary distribution of the Markov chain is

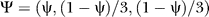
where *ψ* = 1/(1 + 3*s/u*) . The initial state probability of the coal-HMM is given by Ψ. We also investigated more parameter-rich transition probability matrices. In particular, we considered a symmetric model for the transitions between the HC2, HG, and CG states with three parameters *ν*_1_, *ν*_2_, and *ν*_3_, where *ν*_1_ is the probability of making a transition between HC2 and HG, *ν*_2_ the transition probability between HC2 and CG, and *ν*_3_ the transition probability between HG and CG. However, such extended models did not improve the fit significantly with the present amount of available data.

**Figure 6:**
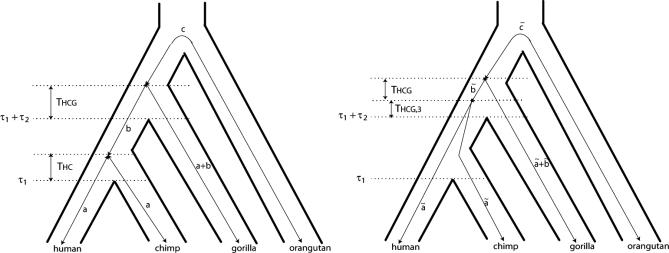
Relation between Parameters in the Coal-HMM and Coalescent-with-Recombination Parameters Left: Coal-HMM and coalescent parameters in state HC1. Right: Coal-HMM and coalescent parameters in state HC2. In both states we assume a molecular clock.

Let **X** = [**X**_1_,…,**X** *_L_*] denote the alignment, consisting of *L* columns (sites) and four rows (corresponding to the four species). The probability *P^e^*(**X** *_i_* | *φ_i_*) that an alignment column **X** *_i_* is emitted from the hidden state *φ_i_* ∈ {HC1,HC2,HG,CG} is determined by the phylogenetic tree corresponding to the hidden state and a substitution rate matrix *Q*. We considered several rate matrices and found that the strand-symmetric rate matrix (e.g., [30])

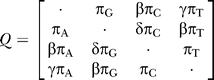
provided a good description of the data. This is perhaps not surprising because the data we analyzed primarily consist of noncoding sequences. The strand-symmetric substitution process has stationary frequencies *π =* (*π*_A_ *,π_G_,π_C_,π*_T_), where *π*_A_ *= π*_T_ and *π*_G_ *= π*_C_. We calibrate the rate matrix such that branch length corresponds to expected substitutions per site. The branch lengths are *a, b,* and *c* in state HC1 and *a~*,*b~*, and *c~* in states HC2, HG, and CG (see Figure 6). We would like to emphasize that continuous time Markov chains take recurrent mutations into account. Furthermore, the so-called CpG effect (higher mutation rates from CpG → TpG and CpG → CpA on the opposite strand) is also taken partially into account because C → T and G → A have particularly high rates in our estimated strand-symmetric rate matrix. For more information on recurrent mutations and CpG hypermutability, refer to Text S2.

Let *η* denote the free parameters in the coal-HMM such that *η* determines the transition matrix *P*(*φ_i_*_-1_, *φ_i_*) = *P_η_*(*φ_i_*_-1_, *φ_i_*), initial state probability vector Ψ = Ψ*_η_*, and emission probabilities 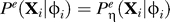 . The joint probability of an alignment **X** and a segmentation *φ = (φ*_1_,…,*φ_L_*) of the genealogies is then given by

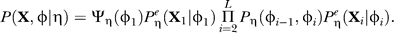



The likelihood 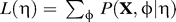 is the sum over all possible segmentations. In the next subsection, we derive the free parameters *η* in the coal-HMM from the coalescent process with recombination.

### Relation between parameters in the coal-HMM and the coalescence process with recombination.

The derivation of the relation between parameters in the coal-HMM and the coalescent process with recombination is carried out in two steps, corresponding to the left and right illustrations in Figure 6. We refer the reader to Chapter 5 in [20] for a thorough description of the coalescence process with recombination and to Yang [16] for a very similar derivation. In the inference procedure we measure all times in expected number of substitutions per site, and then subsequently rescale using an 18-Myr human–orangutan divergence time.

First, consider the situation in the left half of Figure 6. The parameters in the coalescent process are the coalescence time T_HC_ of two given lineages in the HC ancestor, and the coalescence time *T*_HCG_ of two lineages in the HCG ancestor. The two coalescence times *T*_HC_ and *T*_HCG_ are independent and exponentially distributed with means *θ*_HC_ *=* 2*N*_HC_ *μ* and *θ*_HCG_ *=* 2*N*_HCG_ *μ*, where *N*_HC_ and *N*_HCG_ are the effective population sizes in the HC and HCG ancestral populations and *μ* is the mutation rate. The probability that a randomly chosen site belongs to state HC1 is given by

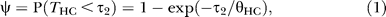
providing a relation between *ψ* and *τ*_2_/θ_HC_. From this observation we also obtain

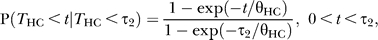
and therefore the average coalescence time for human and chimpanzee in state HC1 is given by

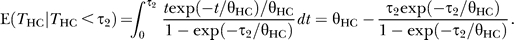



In state HC1, we therefore obtain the following relations between the coal-HMM parameters (*a,b*) and the coalescent-with-recombination parameters (*τ*_1_,*τ*_2_,*θ*_HC_,*θ*_HCG_):

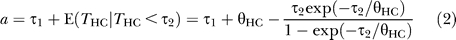


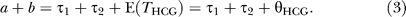



Second, we consider states HC2, HG, and CG. The situation is depicted in the right part of Figure 6 for state HC2. Conditioning on *T*_HC_ *>τ*_2_, the coalescence of any two of the human, chimpanzee, and gorilla sequences is equally likely, and therefore we obtain similar equations as below for states HG and CG. Let *T*_HCG,3_ be the time to coalescence of any two given lineages when three lineages are present in the HCG ancestor. Standard coalescent theory says that *T*_HCG,3_ is exponentially distributed with mean *θ*_HCG_/3. We now obtain the following two equations

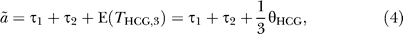


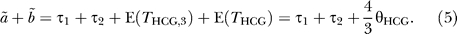



Note that there are five parameters 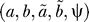 in the coal-HMM, but only four coalescent parameters (*τ*_1_,*τ*_2_,*θ*_HC_,*θ*_HCG_) in equations 1–5. Thus, there is a constraint on the parameters in the coal-HMM. Subtracting equations 4 and 5 we get

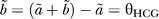
and subtracting equations 3 and 4 we get

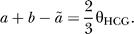
We thus obtain the constraint

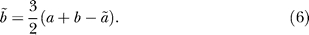



To identify the parameters we solve the system of equations 1–3 and 5. From equation 1 we obtain

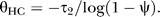
Subtracting equation 3 from 2 and substituting the above we see that

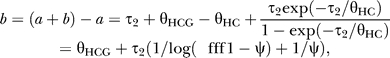
and therefore the parameters in the coalescence process are given by

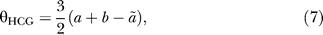


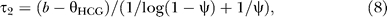


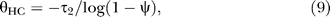


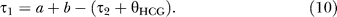



When reporting the parameters, we scale (*a,b,c*) such that ( a + b + c) sums to twice the divergence time between human and orangutan, which is set to 18 Myr. We find the ancestral population sizes by assuming a generation time of 25 y.

### Parameter estimation and standard errors.

We assume the branch lengths fulfil the molecular clock constraint 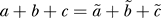 and the relation in equation 6 above. Thus the seven free parameters in the coal-HMM are

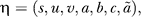
and the maximum likelihood estimates are found using the Baum-Welch method (e.g., [31]). Standard errors for the free parameters are determined from the numerically evaluated Fisher information matrix. Standard errors for functions of the parameters are found using the delta method [32].

### Simulation study.

In order to validate the coal-HMM approximation to the coalescent process with recombination, we conducted a simulation study. The parameters in the simulated coalescent process with recombination are *N*_HC_ = *N*_HCG_ *=* 40,000, *N*_H_ = *N*_C_ *= N*_G_ *=* 30,000, speciation time *τ*_1_ of HC is 4 Myr, speciation time *τ*_2_ of HCG is 5.5 Myr, the time to the ancestor of all four sequences is 18 Myr, the generation time of individuals is 25 y, the length of the sequence is 500,000 bp, and the recombination rate is *r =* 0.0075 (corresponding to a genetic recombination frequency of 1.5 cM per Mb). The simulation from the coalescent-with-recombination process results in a number of recombination events, some of which are visible as change-points along the sequence where the phylogenetic tree changes. We then obtained a sequence alignment where for each position we simulated the evolution of a nucleotide on the phylogenetic tree at that position, and according to the strand-symmetric substitution process. The substitution rate was chosen to match the typical branch lengths in the HCGO quartet, 0.1% change per million years.

Table 3 shows means and standard deviations of the most important quantities for 20 independent simulations. All main quantities are estimated without strong bias. Curiously, *N*_HC_ is estimated with much larger variance than *N*_HCG_, in agreement with analyses of the real data.

**Table 3:**
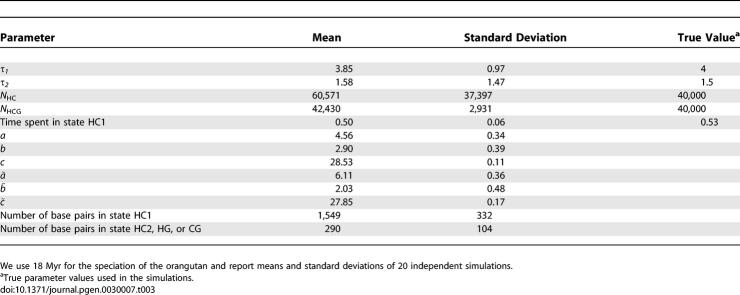
Summary of Simulation Study

**Figure 7:**
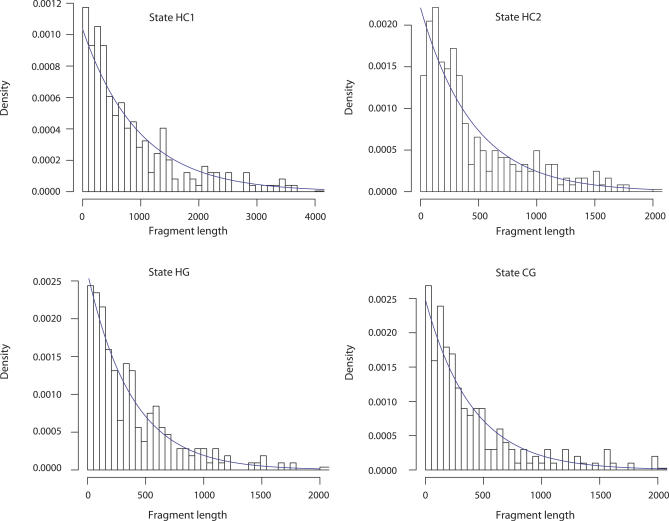
Histogram of Fragment Lengths for the Four Different Genealogies The distribution of fragment lengths is reasonably well approximated by the geometric distribution (blue line). This property is a basic assumption of the coal-HMM.

One of the assumptions of the coal-HMM is that the distribution of fragment lengths for each hidden state is geometric. Considering simulations from a coalescence with recombination process, the fragments for the coal-HMM are aggregations of fragments with branch lengths corresponding to the same state of the coal-HMM. Figure 7 shows the distribution of aggregated fragment sizes from a coalescent-with-recombination simulation. Although the coalescent-with-recombination process is non-Markovian when viewed as a process along the sequence [33], the fragment lengths are reasonably well-described by a geometric distribution. The slight deficiency of very short fragments in Figure 7 is due to the aggregation of fragments with correlated coalescence times that occurs for each state.

**Figure 8:**
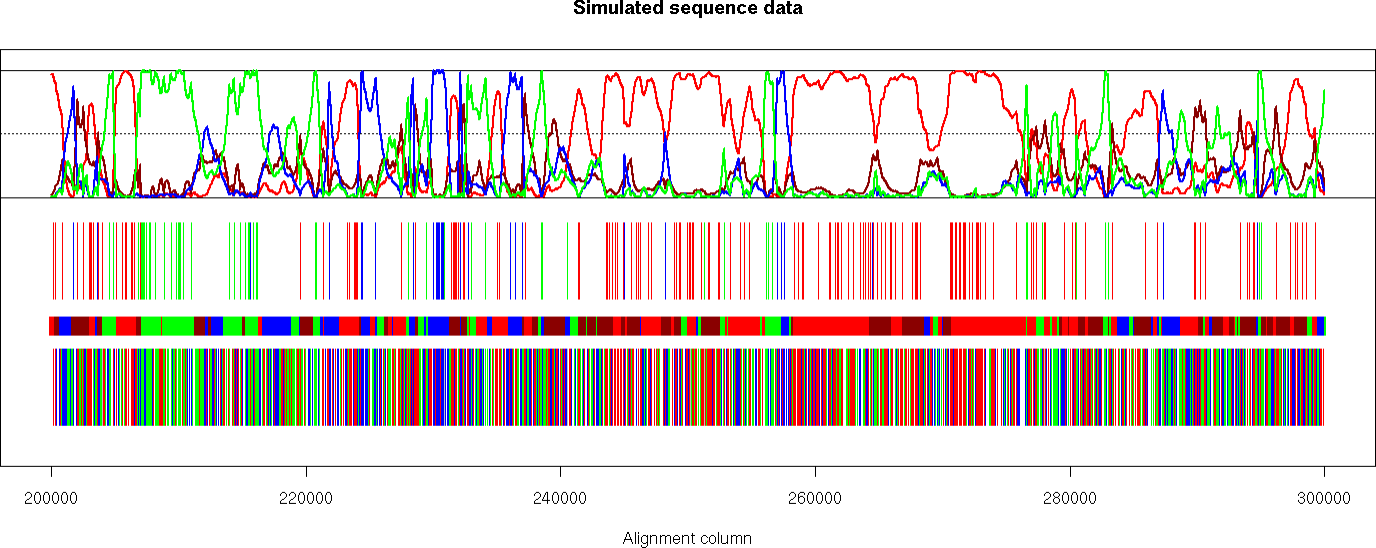
Inferred Genealogies from Simulated Data (Bottom) Site information without outgroup: sites shared by HC in red, by HG in blue, and by CG in green (see first, third, and last columns in Table 1). (Second from bottom) True genealogical state in simulations: state HC1 in red, HC2 in dark red, HG in blue, and CG in green. (Third from bottom) Site information with outgroup: sites supporting state HC1 or HC2 in red, HG in blue, and CG in green (see second, third, and last columns in Table 1). (Top) Posterior probabilities for the four states. Coloring as in second from bottom.

Figure 8 compares simulated and inferred genealogies from a segment of 100,000 base pairs from one of the simulation runs. Coloring corresponds to the different states of the Markov chain (see Table 1), and the upper panel shows the posterior probability of each of the states. We see long fragments where state HC1 is the true state, and we see regions where the true state changes frequently. This is caused by the coalescence time for states HC2, HG, and CG being further back in time than the coalescence time for state HC1, leaving more time for recombination events to accumulate and cause more frequent changes of genealogy. This phenomenon is also reflected in the reconstruction, i.e., in the posterior probabilities, where we see good agreement between true and inferred genealogical states of the long fragments where state HC1 is the true state, and we also see that areas where the true states change frequently are inferred as such. However, in addition, we see that within regions where the true states change frequently, the reconstruction in general is quite uncertain.

### Data.

Chimpanzee–gorilla–orangutan sequence data from Targets 1 (Chromosome 7), 106 (Chromosome 20), 121 (Chromosome 2), and 122 (Chromosome 20) were obtained from the NIH Intramural Sequencing Center Web site (http://www.nisc.nih.gov), and the corresponding human sequences were downloaded from GenBank (http://www.ncbi.nlm.nih.gov/Genbank). For each target, sequences from the four species were aligned using the MAVID alignment software [34]. The resulting alignment was manually inspected, and columns corresponding to an insertion in orangutan were removed. Next, the alignment was subdivided in cases where more than 50 deletions were present in one of the species, and finally, alignment columns with gaps were removed. For the X-chromosome, the alignment used by [2] was downloaded and filtered for poorly aligned segments (more than five segregating sites in 20 base pair windows). Fine-scale (from HapMap data [24]) and region-wide (from pedigree data [25]) estimates of the recombination rate were downloaded from the University of California Santa Cruz genome browser (http://www.genome.ucsc.edu).

## Supporting Information

Figure S1 Analysis of 1,255 kb from Target 1 (204 KB PDF)

Figure S2 Analysis of 258 kb from Target 106 (50 KB PDF)

Figure S3 Analysis of 231 kb from Target 121 (52 KB PDF)

Figure S4 Analysis of 92 kb from Target 122 (26 KB PDF)

Figure S5 Analysis of 263 kb from Target 46 (26 KB PDF)

Text S1 Correlation between Transitions and Fine-Scale Recombination Rates (57 KB PDF)

Text S2 Discussion of Recurrent Mutations and CpG Hypermutability (291 KB PDF)

## Abbreviations

CG: chimp–gorilla
coal-HMM: coalescent hidden Markov model
HC: human–chimp
HCG: human–chimp–gorilla
HCGO: human–chimp–gorilla–orangutan
HG: human–gorilla
HMM: hidden Markov model
Myr: million years

## References

[pgen-0030007-b001] Enard W, Pääbo S (2004). Comparative primate genomics. Annu Rev Genomics Hum Genet.

[pgen-0030007-b002] Patterson N, Richter DJ, Gnerre S, Lander ES, Reich D (2006). Genetic evidence for complex speciation of human and chimpanzees. Nature.

[pgen-0030007-b003] Osada N, Wu CI (2005). Inferring the mode of speciation from genomic data: A study of the great apes. Genetics.

[pgen-0030007-b004] Innan H, Watanabe H (2006). The effect of gene flow on the coalescent time in the human-chimpanzee ancestral population. Mol Biol Evol.

[pgen-0030007-b005] Barton NH (2006). Evolutionary biology: How did the human species form?. Curr Biol.

[pgen-0030007-b006] Nielsen R, Bustamante C, Clark AG, Glanowski S, Sackton TB (2005). A scan for positively selected genes in the genomes of humans and chimpanzees. PLoS Biol.

[pgen-0030007-b007] Cela-Conde CJ, Ayala FJ (2003). Genera of the human lineage. Proc Natl Acad Sci U S A.

[pgen-0030007-b008] Senut B, Pickford M, Gommery D, Mein P, Cheboi K (2001). First hominid from the Miocene (Lukeino Formation, Kenya). C R Acad Sci II.

[pgen-0030007-b009] Haile-Selassie Y (2001). Late Miocene hominoids from the Middle Awash, Ethiopia. Nature.

[pgen-0030007-b010] Brunet M, Guy F, Pilbeam D, Mackaye HT, Likius A (2002). A new hominid from the Upper Miocene of Chad, Central Africa. Nature.

[pgen-0030007-b011] Brunet M, Guy F, Pilbeam D, Lieberman DE, Likius A (2005). New material of the earliest hominid from the Upper Miocene of Chad. Nature.

[pgen-0030007-b012] Wolpoff MH, Senut B, Pickford M, Hawks J (2002). Palaeoanthropology. *Sahelanthropus* or *‘Sahelpithecus'*?. Nature.

[pgen-0030007-b013] Chen FC, Li WH (2001). Genomic divergences between humans and other hominoids and the effective population size of the common ancestor of humans and chimpanzees. Am J Hum Genet.

[pgen-0030007-b014] Wall JD (2003). Estimating ancestral population sizes and divergence times. Genetics.

[pgen-0030007-b015] O'hUigin C, Satta Y, Takahata N, Klein J (2002). Contribution of homoplasy and of ancestral polymorphism to the evolution of genes in anthropoid primates. Mol Biol Evol.

[pgen-0030007-b016] Yang ZH (2002). Likelihood and Bayes estimation of ancestral population sizes in hominoids using data from multiple loci. Genetics.

[pgen-0030007-b017] Rannala B, Yang Z (2003). Bayes estimation of species divergence times and ancestral population sizes using DNA sequences from multiple loci. Genetics.

[pgen-0030007-b018] Satta Y, Hickerson M, Watanabe H, O'Huigin C, Klein J (2004). Ancestral population sizes and species divergence times in the primate lineage on the basis of intron and BAC end sequences. J Mol Evol.

[pgen-0030007-b019] Mikkelsen TS, Hillier LW, Eichler EE, Zody MC, Jaffe DB (2005). Initial sequence of the chimpanzee genome and comparison with the human genome. Nature.

[pgen-0030007-b020] Hein J, Scheirup MH, Wiuf C (2005). Gene genealogies, variation and evolution: A primer in coalescence theory.

[pgen-0030007-b021] Yang Z (1995). A space–time process model for the evolution of DNA sequences. Genetics.

[pgen-0030007-b022] Felsenstein J, Churchill G (1996). A hidden Markov model approach to variation among sites in rate of evolution. Mol Biol Evol.

[pgen-0030007-b023] Siepel A, Haussler D, Nielsen R (2005). Phylogenetic hidden Markov models. Statistical methods in molecular evolution.

[pgen-0030007-b024] Myers S, Bottolo L, Freeman C, McVean G, Donnelly P (2005). A fine-scale map of recombination rates and hotspots across the human genome. Science.

[pgen-0030007-b025] Kong A, Gudbjartsson DF, Sainz J, Jonsdottir GM, Gudjonsson SA (2002). A high-resolution recombination map of the human genome. Nat Genet.

[pgen-0030007-b026] Kumar S, Filipski A, Swarna V, Walker A, Hedges SB (2005). Placing confidence limits on the molecular age of the human–chimpanzee divergence. Proc Natl Acad Sci U S A.

[pgen-0030007-b027] Glazko GV, Nei M (2003). Estimation of divergence times for major lineages of primate species. Mol Biol Evol.

[pgen-0030007-b028] Sarich VM, Wilson AC (1973). Generation time and genomic evolution in primates. Science.

[pgen-0030007-b029] Siepel A, Haussler D (2004). Combining phylogenetic and hidden Markov models in biosequence analysis. J Comput Biol.

[pgen-0030007-b030] Yap VB, Speed TP (2004). Modeling DNA base substitution in large genomic regions from two organisms. J Mol Evol.

[pgen-0030007-b031] Ewens WJ, Grant GR (2001). Statistical methods in bioinformatics.

[pgen-0030007-b032] Oehlert GW (1992). A note on the delta method. Am Stat.

[pgen-0030007-b033] Wiuf C, Hein J (1999). Recombination as a point process along sequences. Theor Popul Biol.

[pgen-0030007-b034] Bray N, Pachter L (2004). MAVID: Constrained ancestral alignment of multiple sequences. Genome Res.

