# Supplementary figures and images for: Genomic Relationships and Speciation Times of Human, Chimpanzee, and Gorilla Inferred from a Coalescent Hidden Markov Model
Source: PLoS Genet. 2007 Feb 23;3(2):e7. doi: 10.1371/journal.pgen.0030007 (PMC1802818; doi:10.1371/journal.pgen.0030007)

Figure S1: Analysis of 1255 kb from target1

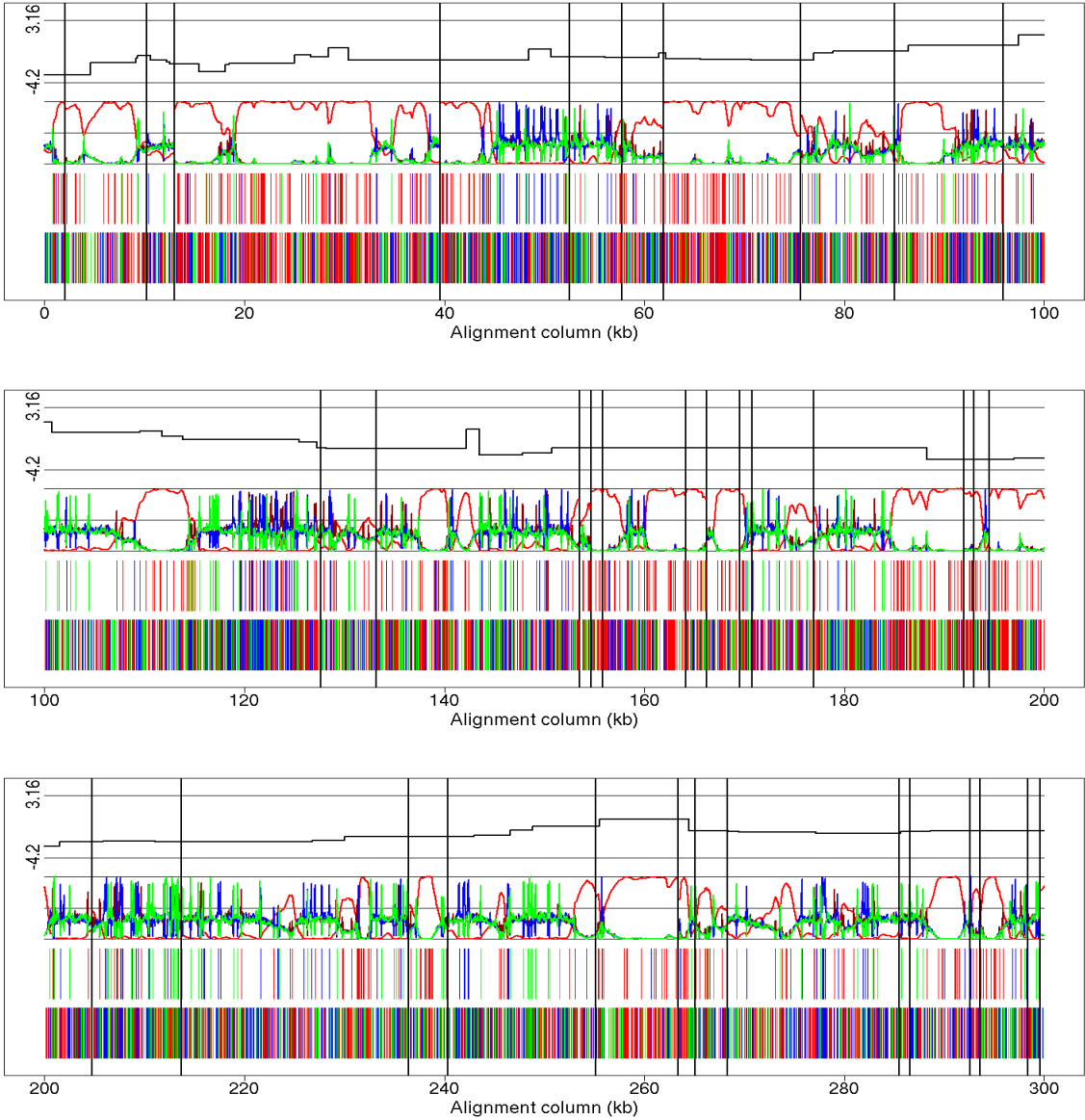

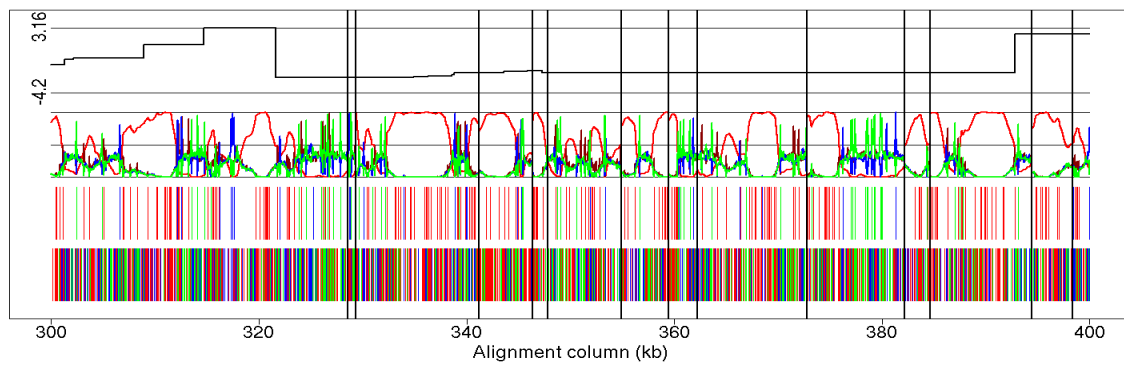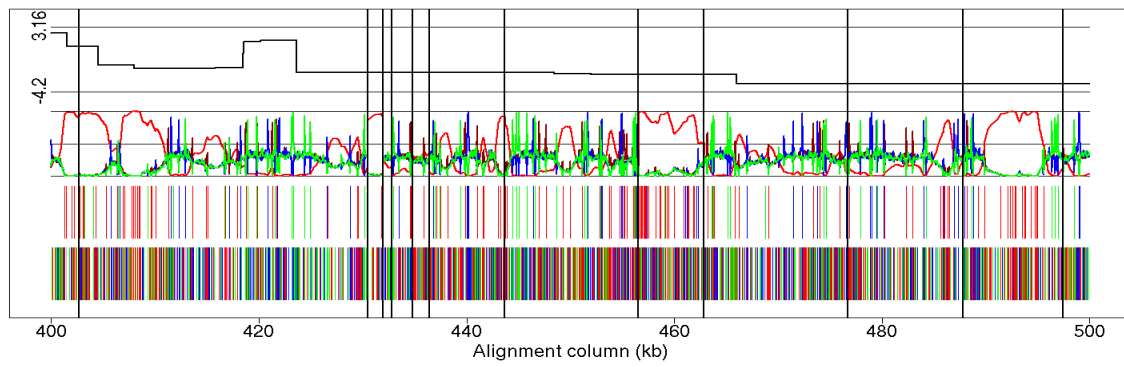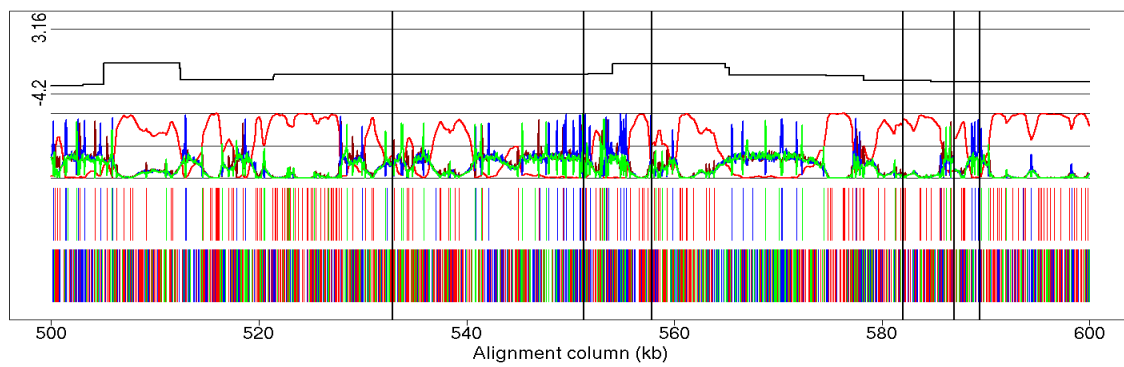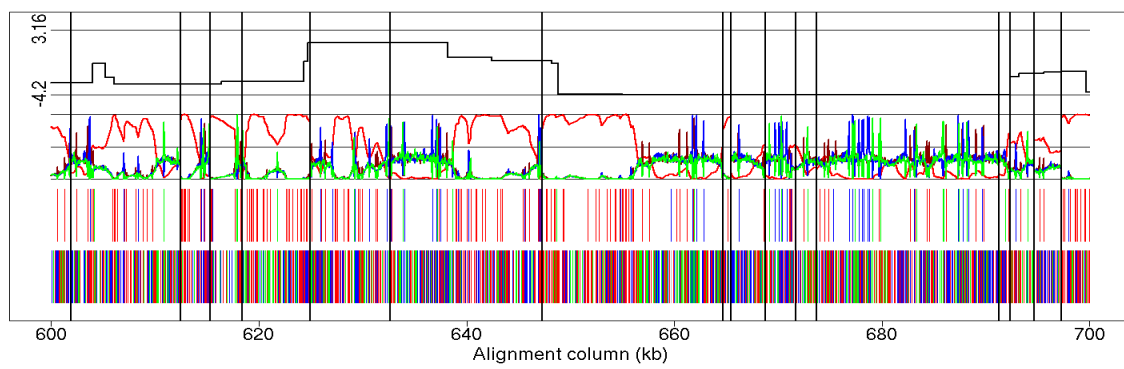

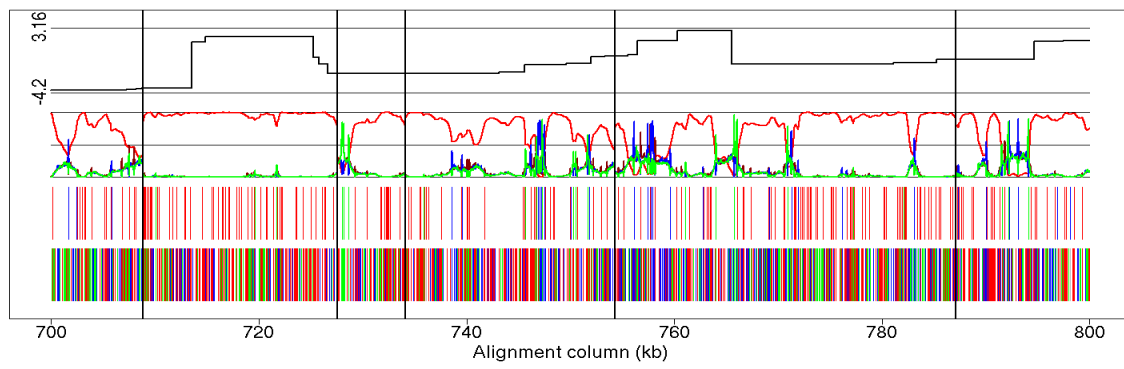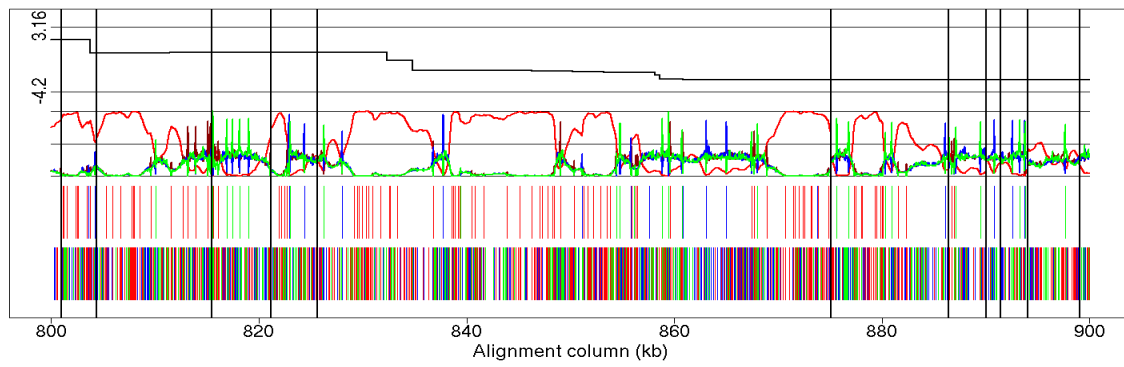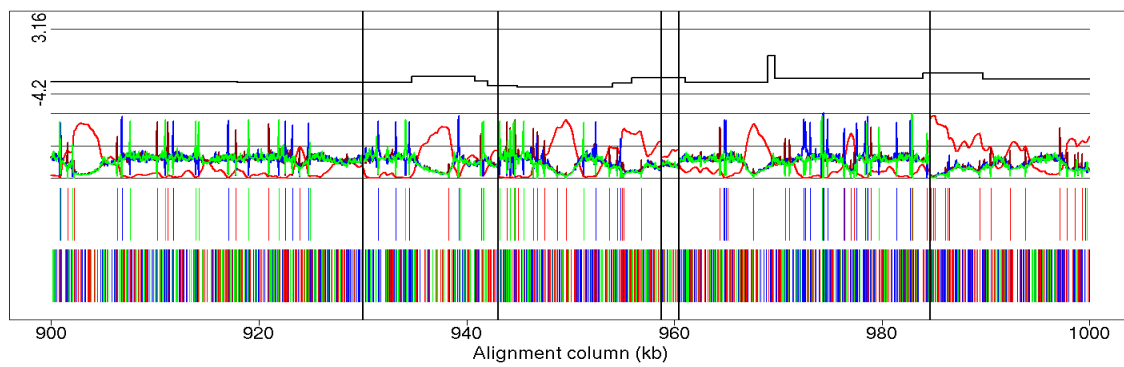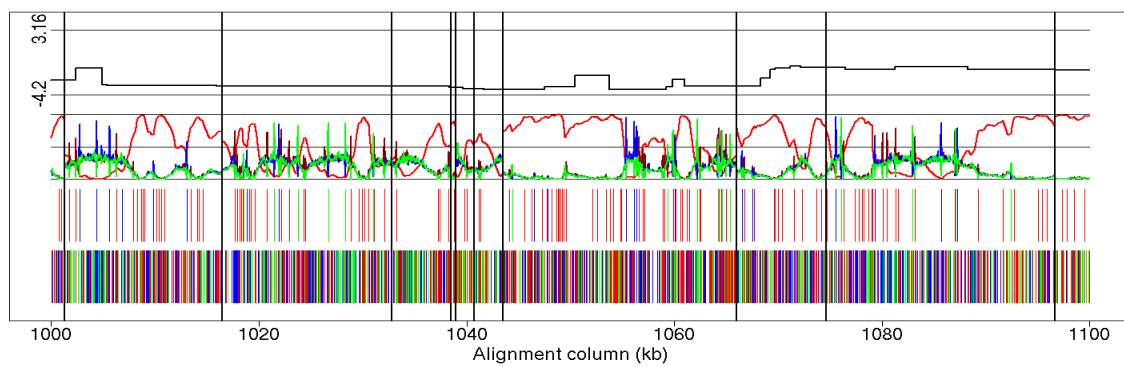

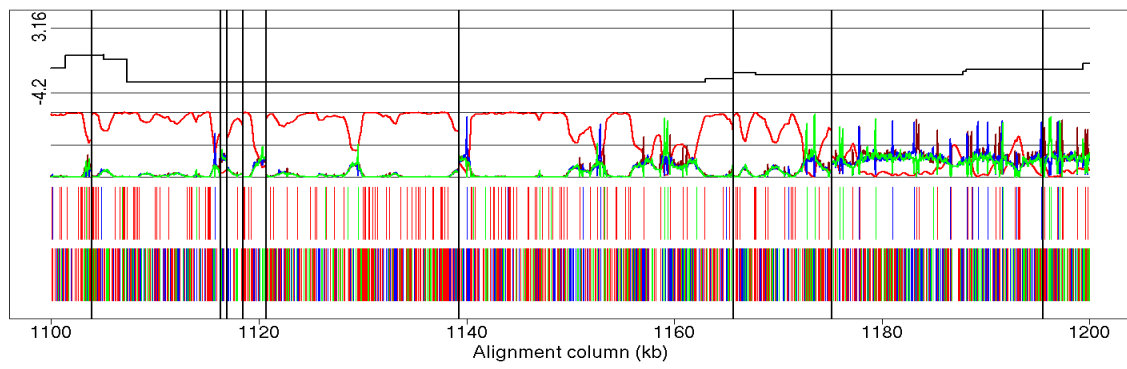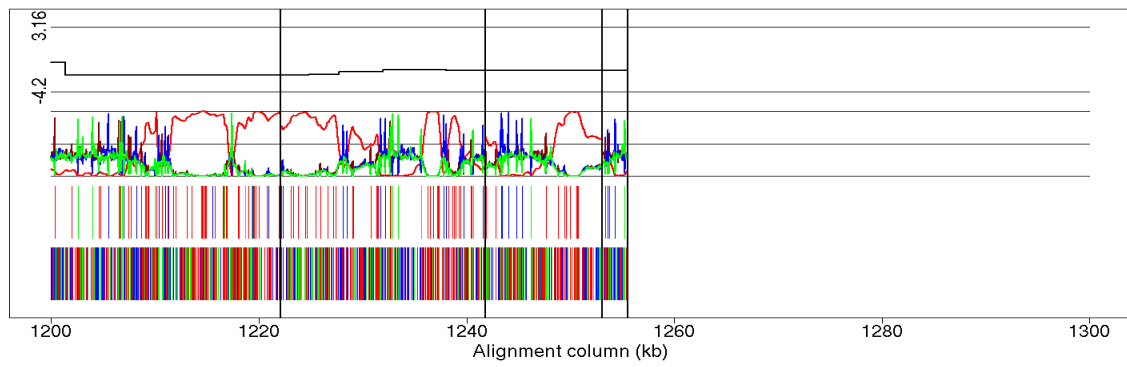

Supplement: Figure S1 — (204 KB PDF) [file pgen.0030007.sg001.pdf]

**Figure S2: Analysis of 258 kb from target106**

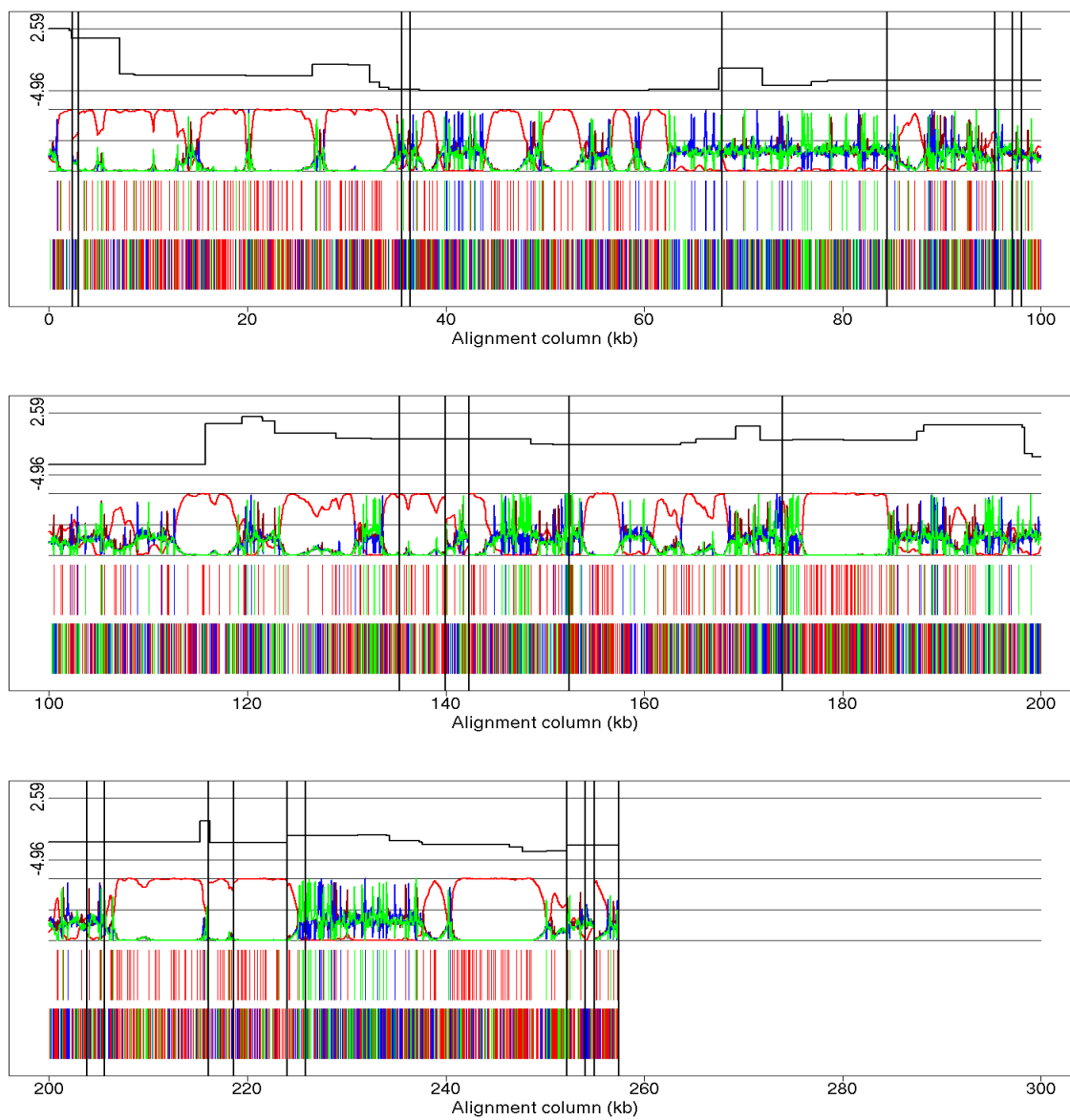

Supplement: Figure S2 — (50 KB PDF) [file pgen.0030007.sg002.pdf]

Figure S3: Analysis of 231 kb from target121

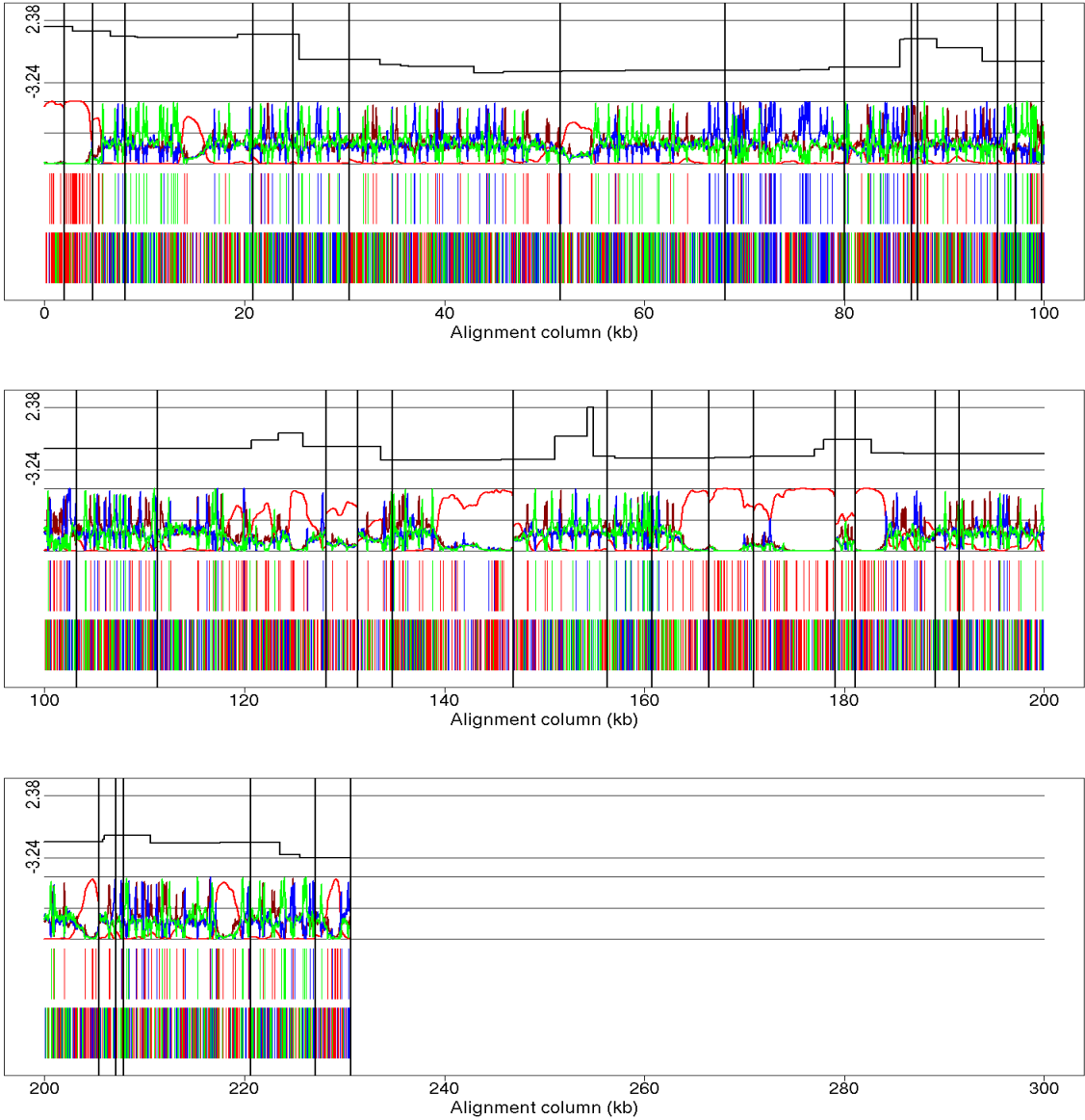

Supplement: Figure S3 — (52 KB PDF) [file pgen.0030007.sg003.pdf]

Figure S4: Analysis of 92 kb from target122

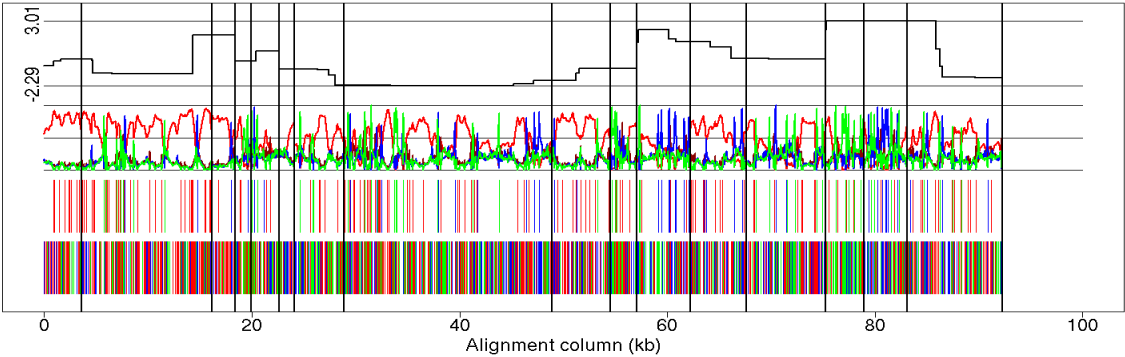

Supplement: Figure S4 — (26 KB PDF) [file pgen.0030007.sg004.pdf]

Figure S5: Analysis of 263 kb from target46 (X-chromosome)

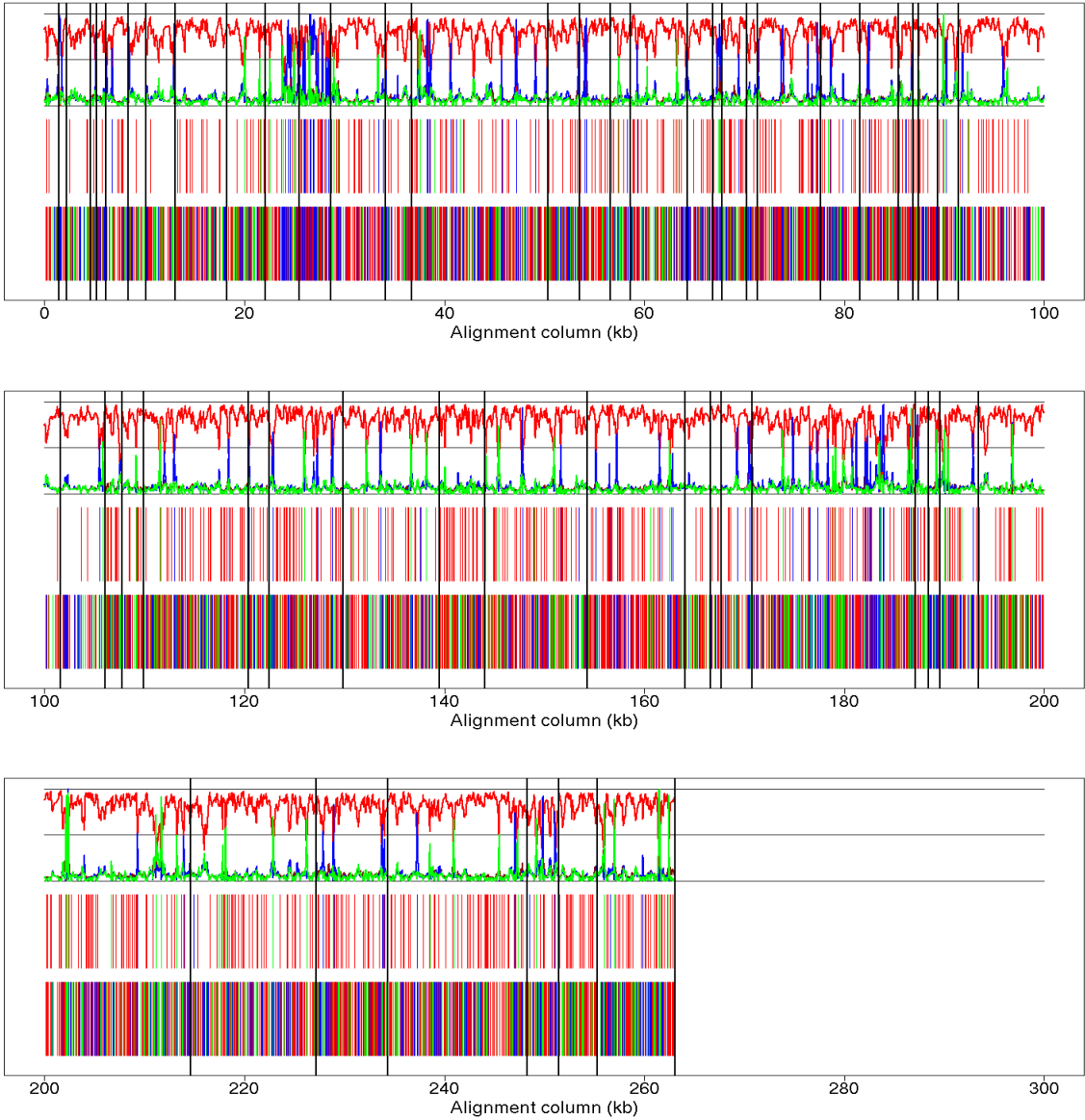

Supplement: Figure S5 — (26 KB PDF) [file pgen.0030007.sg005.pdf]
