# Supplementary material for: Genomic Relationships and Speciation Times of Human, Chimpanzee, and Gorilla Inferred from a Coalescent Hidden Markov Model
Source: PLoS Genet. 2007 Feb 23;3(2):e7. doi: 10.1371/journal.pgen.0030007 (PMC1802818; doi:10.1371/journal.pgen.0030007)
Supplement: Text S1 — (57 KB PDF) [file pgen.0030007.sd001.pdf]

Supporting Information Text S1: Correlation between transitions and fine-scale recombination rates.

The fine-scale recombination rate from human diversity data [1] has been estimated at every point along the alignment. In order to determine if the fine-scale recombination rate can explain the pattern of transitions between different topologies, we considered points along Target 1 separated by 500 base pairs. Firstly, a genealogy at each point is determined from the value of the maximum posterior probability. Secondly, the transitions from one point to the next is divided into four categories determined by

a Both points are in the HC1 genealogy.

b The first point is in the HC1 genealogy and the second is not in the HC1 genealogy.

c The first point is not in the HC1 genealogy and the second point is in the HC1 genealogy.

d Neither point is in the HC1 genealogy.

We also divided the fine-scale recombination rates into three categories; high, medium or low.

Using these criteria, we end up with the following contingency table:

	a	b	c	d
high	360	44	47	176
medium	642	96	95	345
low	357	60	61	226

We tested if the frequencies in the rows differed significantly and could not accept the hypothesis (X-squared=6.379, df=6, p-value = 0.3821).

The above analysis was repeated for different numbers of seperating base pairs (250 and 1,000 instead of 500), and in those cases we also did not observe different patterns of transitions depending on the fine-scale recombination rate.

References

[1] Myers S, Bottolo L, Freeman C, McVean G, Donnelly P (2005) A fine-scale map of recombination rates and hotspots across the human genome. Science 310:321–324.
