# Supplementary material for: Genomic Relationships and Speciation Times of Human, Chimpanzee, and Gorilla Inferred from a Coalescent Hidden Markov Model
Source: PLoS Genet. 2007 Feb 23;3(2):e7. doi: 10.1371/journal.pgen.0030007 (PMC1802818; doi:10.1371/journal.pgen.0030007)

Supporting Information Text S2: Discussion of recurrent mutations and CpG hypermutability.

The purpose of Supporting Information Text S2 is to discuss recurrent mutations and CpG hypermutability in more detail.

Firstly, we would like to emphasize that continuous time Markov chains on phylogenetic trees allow recurrent mutations. Furthermore, the CpG effect (higher mutation rates from CpG→TpG and CpG→CpA on the opposite strand) is partially taken into account because C→T and G→A have particularly high rates in our estimated strand-symmetric rate matrix which is given by

	A	G	C	T
A	-1.08	0.79	0.20	0.09
G	0.52	-0.95	0.30	0.13
C	0.13	0.30	-0.95	0.52
T	0.09	0.20	0.79	-1.07

Note that among the rates from C to A,G or T, it is the rate to T that has the largest value, while among the rates away from G to A,C or T, it is the rate to A that has the largest value.

Secondly, while it is true that sites that strongly support a particular topology are the most informative, there is also much information from the less informative sites. In Table 1, we have summarized the sites that strongly *and* weakly support a particular topology.

HCGO	Topology
110x	HC
101x	HG
011x	CG

Table 1: Sites supporting a particular topology (x denotes any base). When x=0, the topology is strongly supported, while if x≠0, the topology is weakly supported.

The reason why a site such as HCGO=AGAT provides information about topology is that the probability of observing a specific alignment column in our model depends on the underlying phylogenetic tree. Therefore sites such as AGAA, AGAT and AGAC carry information about the topology (they all support the HG topology), although of course a AGAG site more strongly supports the HG topology.

We investigated the effect of the two above mentioned issues by carrying out a coal-HMM analysis on three new data sets. The three new data sets were constructed as follows. Firstly, we re-aligned the largest data set in our study, Target 1, with Gibbon as a fifth species. This new aligment constitutes the first data set. Secondly, we filtered out all putative CpG mutations (this alignment is referred to as CpG-corr), and finally we filtered out all sites where the site patterns suggest that at least two mutations must

have occured (this alignment is referred to as Rec-CpG-corr). The first filtering removes the CpG hypermutability effect, and the second filtering removes (some of the) effects due to recurrent mutations in hypermutable sites. Removing all these sites is likely to be an overcorrection, but our emphasis here is mainly to investigate the robustness of our coal-HMM to effects due to recurrent mutations.

A summary of the site patterns in the three new data sets is given in Table 2.

HCGOM	Topology	Original	CpG-corr	Rec-CpG-corr
00001		25848	22050	22050
00010		20150	17283	17283
00011		10264	8435	8435
00100		10464	9132	9132
00101		370	176	0
00110	HC	323	157	0
00111	HC	1762	1376	1376
01000		6472	5423	5432
01001		226	86	0
01010	HG	185	77	0
01011	HG	312	186	186
01100	CG	310	180	180
01101	CG	168	68	0
01110		276	131	0
01111		6365	5399	5399

Table 2: Summary of informative sites in the three new data sets. In the first column, M refers to Gibbon, and the human allele is defined to have state 0. In the second column, the supported topology is only indicated when it is strongly supported.

Counting strongly supportive sites only, we obtain Table 3. Table 3 suggests a major effect of both CpG hypermutability and recurrent mutations. Indeed, from these counts it seems that the ratios of being in a HC:HG:CG topology is roughly 4:1:1 for the uncorrected data set, 6:1:1 for the CpG-corrected data set and 7:1:1 for the recurrent-CpG-corrected data set. However, these calculations are based on strongly informative sites only and, as described above, other sites also carry information about topology. By taking all sites into account we get a much different picture (see below). This is perhaps not surprising since the strongly informative sites only constitute a fraction of the informative sites.

We analysed the three new data sets (after removing Gibbon) using our coal-HMM software. Parameter estimates are summarized in Table 4 and Table 5 and show a remarkable robustness to both the CpG-correction and the recurrent-mutations-correction. From Table 4 we see that the time spent in the basic state HC1 is only slightly changed after corrections, although the mean fragment length is much longer after corrections. From Table 5 we conclude that the speciation times and divergence times do have an

HCGOM	Topology	Original	CpG-corr	Rec-CpG-corr
0011x	HC	2085	1533	1376
0101x	HG	497	263	186
0110x	CG	478	248	180

Table 3: Counts of sites that strongly support a specific topology.

impact, but not as strong as one might perhaps expect. The corrections have a larger impact on ancestral population sizes, but these quantities are determined with large standard errors in the first place (recall Figure 4 in the manuscript), and therefore we conclude that the coal-HMM is rather robust to effects due to CpG hypermutability and recurrent mutations. The best option for future research is of course to explicitly treat these cases in the model, but such an approach requires a better understanding of context dependent substitution processes and is beyond the scope of the analysis.

	time spent in state		mean # bp in state	
	HC1	HC2, HG or CG	HC1	HC2, HG or CG
Original	0.47	0.17	2645	63
CpG-corr	0.48	0.17	3663	66
Rec-CpG-corr	0.52	0.16	5464	68

Table 4: Time spent in basic and alternative states, and mean average length in the basic and alternative states.

	τ_1 (Myr)	τ_2 (Myr)	N_{HC}	N_{HCG}
Original	4.0	2.3	72000	38000
CpG-corr	3.6	3.1	94000	25000
Rec-CpG-corr	3.7	3.2	87000	19000

	a (Myr)	b (Myr)	$\tilde{a}$ (Myr)	$\tilde{b}$ (Myr)
Original	5.0	3.2	6.9	1.9
CpG-corr	5.0	3.0	7.1	1.2
Rec-CpG-corr	5.1	2.7	7.2	1.0

Table 5: Speciation times (τ_1, τ_2), effective ancestral human-chimp and human-chimp-gorilla population sizes (N_{HC}, N_{HCG}), and divergence times ($a, b, \tilde{a}, \tilde{b}$) for uncorrected data, after filtering out hypermutable CpG sites and after filtering out both CpG sites and recurrent mutations.

We further investigated the impact of CpG hypermutability and recurrent mutations by considering plots of site patterns and posterior probabilities. The results for the first 500 kb is shown in Figures 1-5 below and further support our conclusion that the coal-HMM is rather robust to CpG and recurrent mutation effects. If these plots are studied

closely, it is evident that strongly informative sites supporting an alternative state does not always lead to a decrease in posterior probability of the alternative state. It is also quite clear why the average length in the HC state is sensitive to removal of these sites, but we do not make a strong case for this length except when comparing the different targets.

Figure 1: Analysis of the first 100kb from the uncorrected (top), `CpG`-corrected (middle) and recurrent-`CpG`-corrected alignments (bottom).

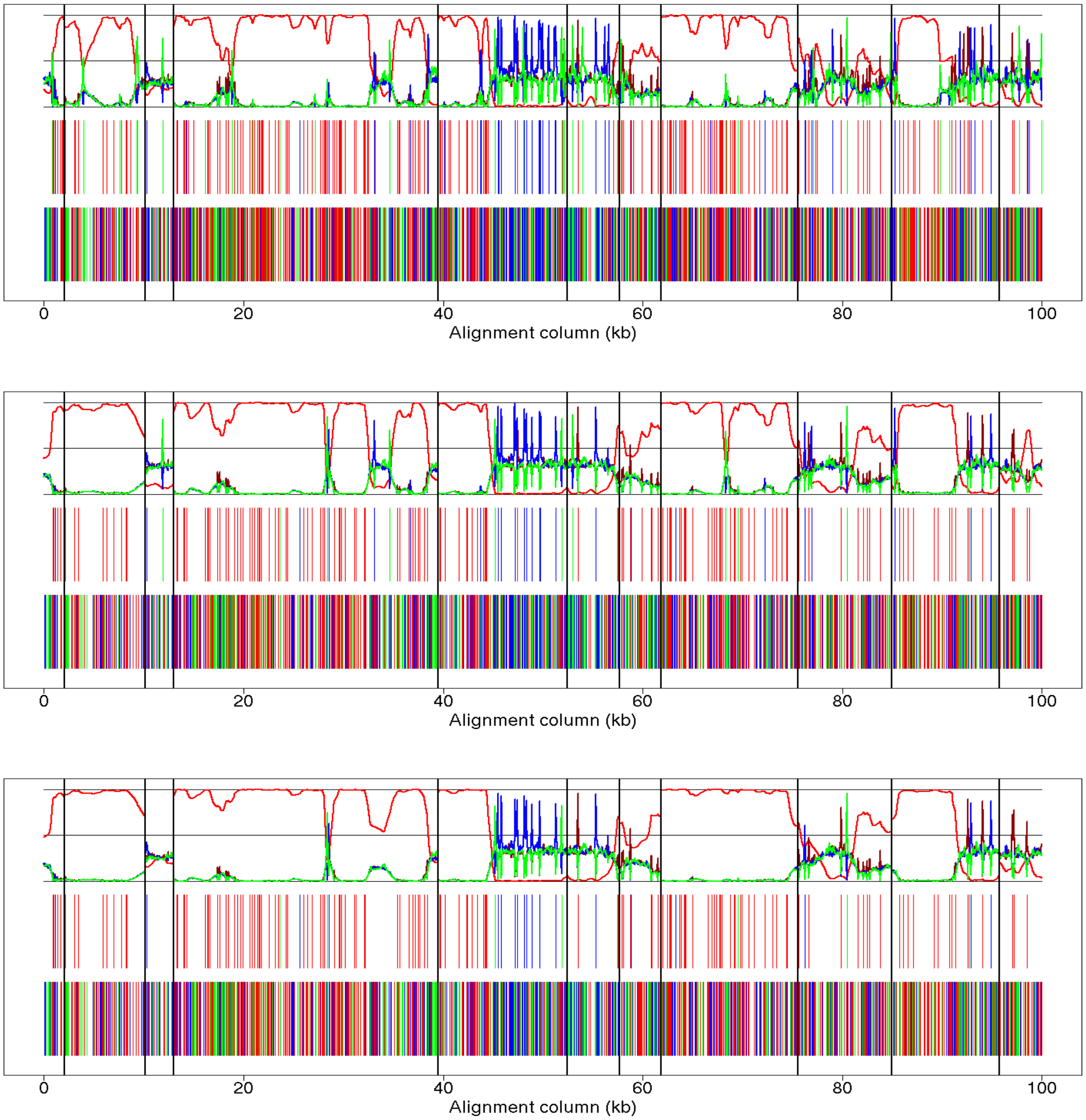

Figure 2: Analysis of positions 100-200kb from the uncorrected (top), `CpG`-corrected (middle) and recurrent-`CpG`-corrected alignments (bottom).

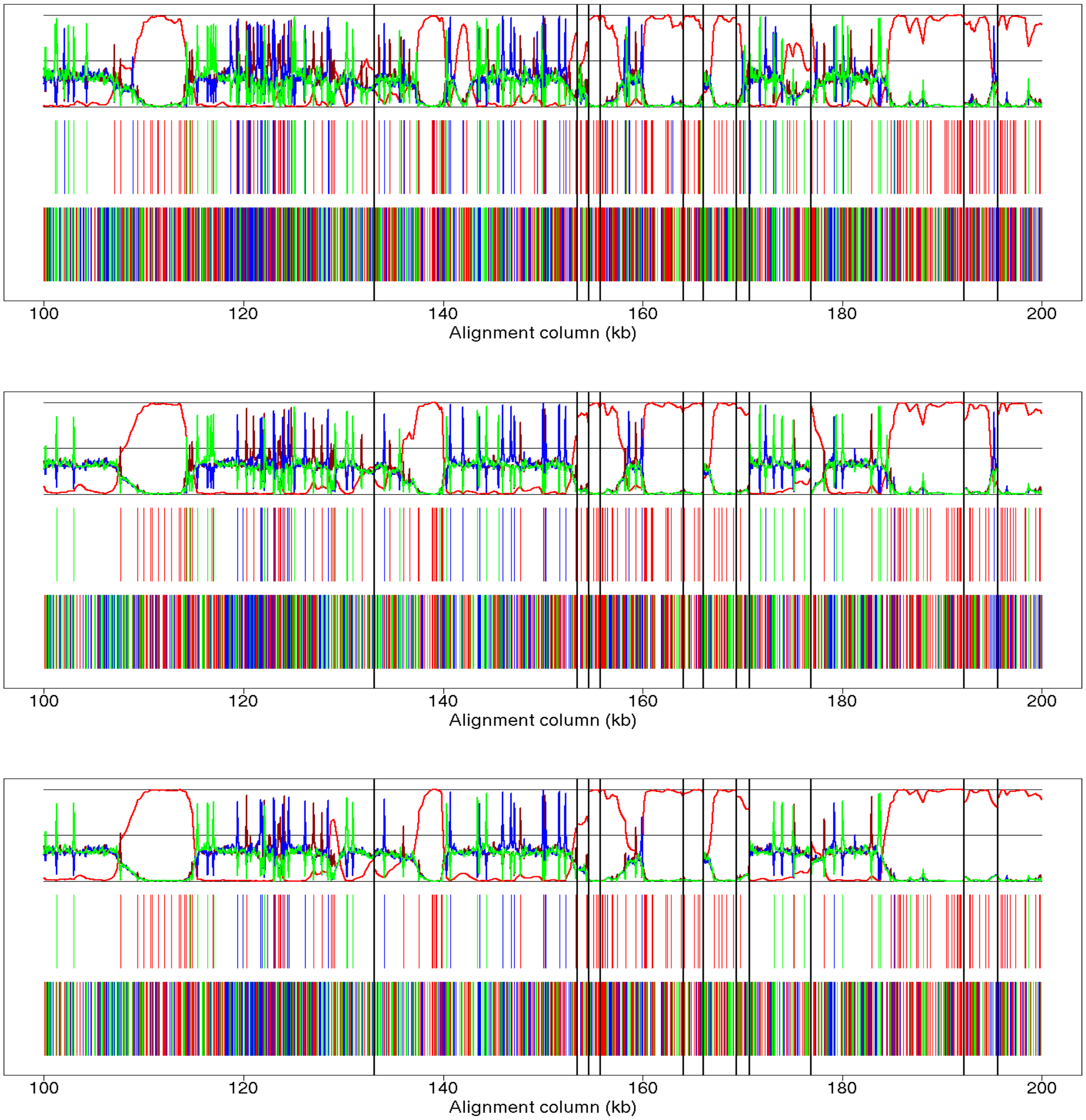

Figure 3: Analysis of positions 200-300kb from the uncorrected (top), `CpG`-corrected (middle) and recurrent-`CpG`-corrected alignments (bottom).

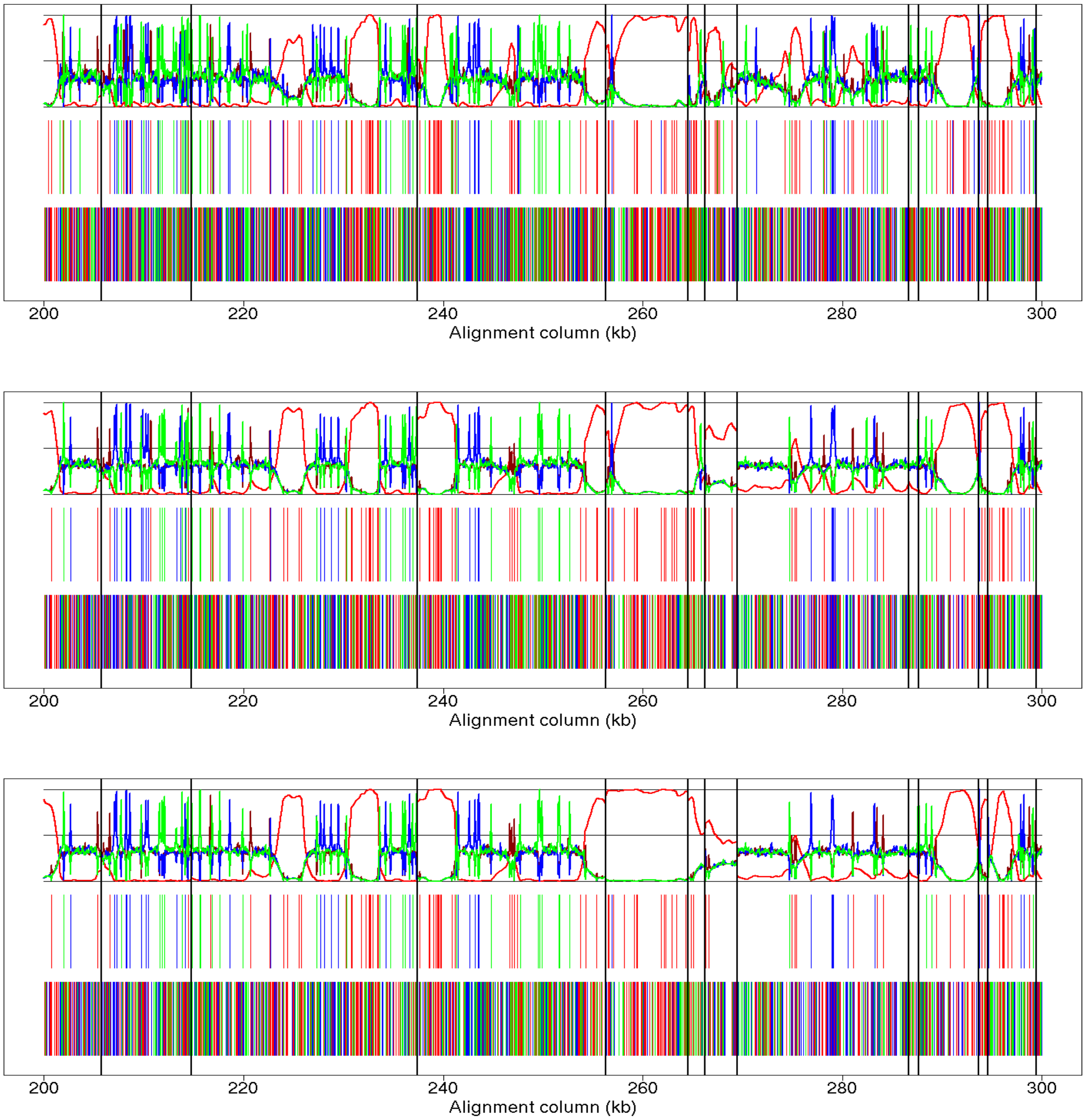

Figure 4: Analysis of positions 300-400kb from the uncorrected (top), CpG-corrected (middle) and recurrent-CpG-corrected alignments (bottom).

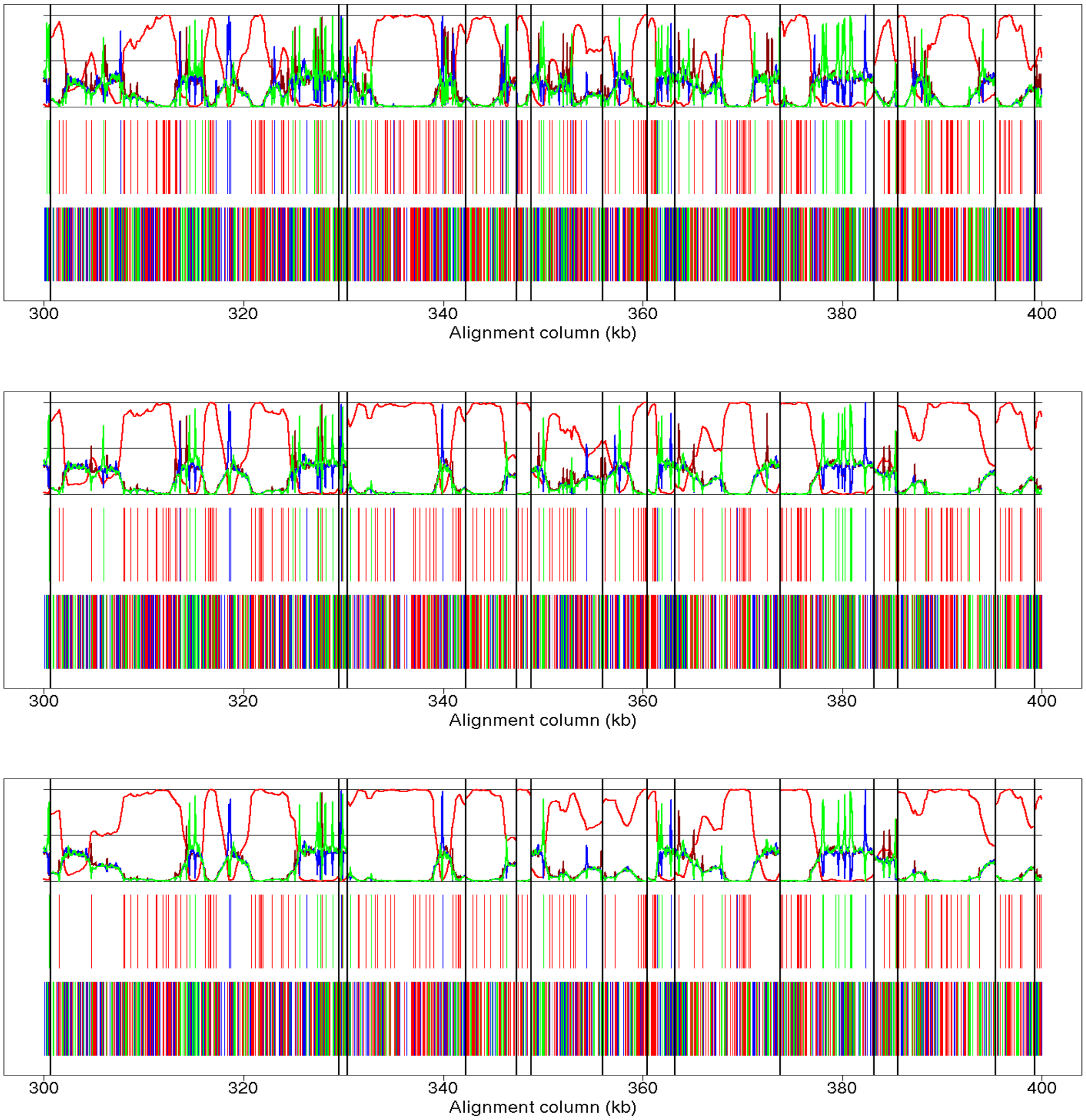

Figure 5: Analysis of positions 400-500kb from the uncorrected (top), CpG-corrected (middle) and recurrent-CpG-corrected alignments (bottom).

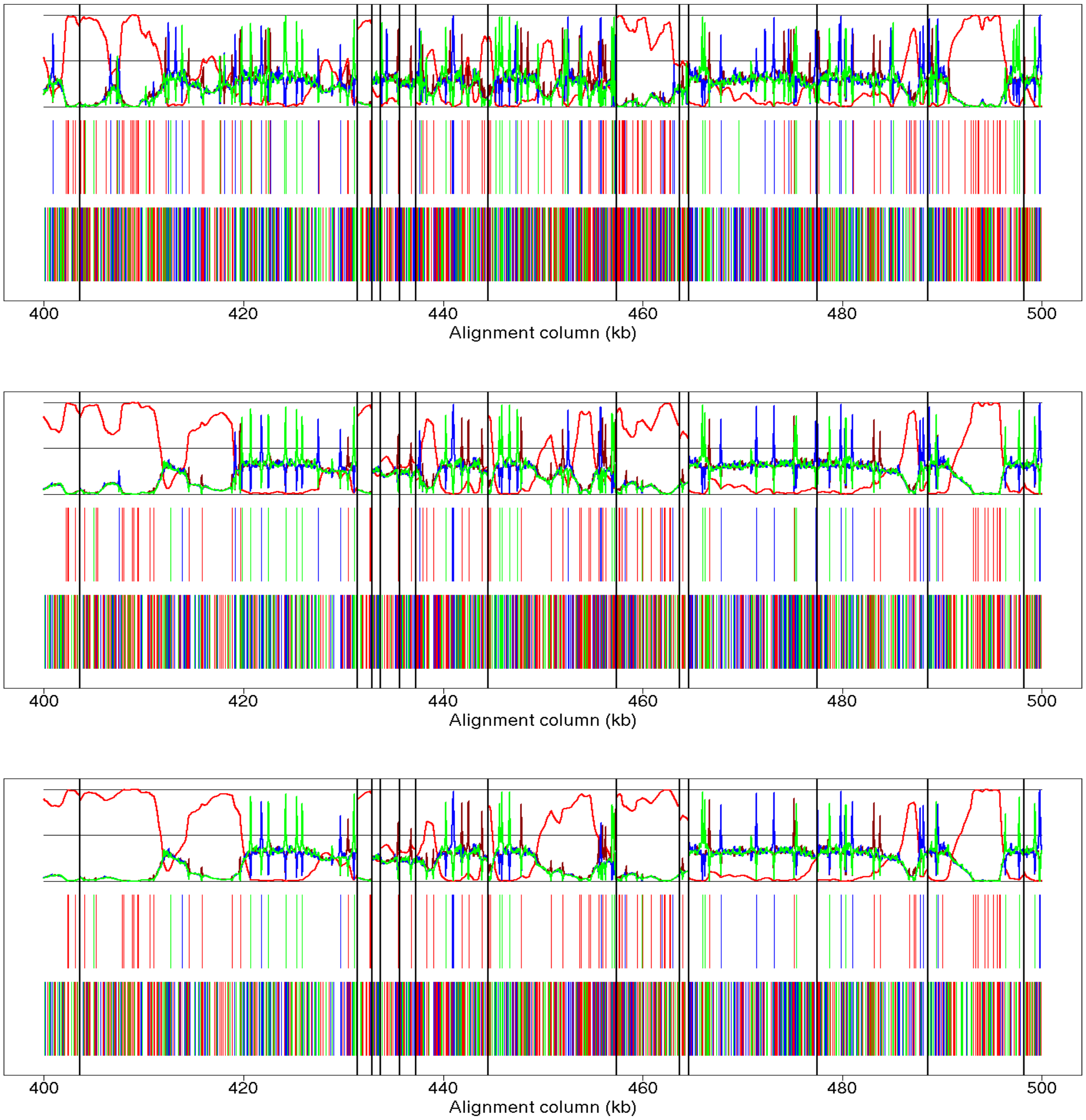

Supplement: Text S2 — (291 KB PDF) [file pgen.0030007.sd002.pdf]
